# Synthetic strategies toward 1,3-oxathiolane nucleoside analogues

**DOI:** 10.3762/bjoc.17.182

**Published:** 2021-11-04

**Authors:** Umesh P Aher, Dhananjai Srivastava, Girij P Singh, Jayashree B S

**Affiliations:** 1 Chemical Research Department, Lupin Research Park, Lupin Limited, 46A/47A, Village Nande, Taluka Mulshi, Pune-412115, Maharashtra, India; 2 Department of Pharmaceutical Chemistry, Manipal College of Pharmaceutical Sciences, Manipal Academy of Higher Education, Manipal-576104, Karnataka, India

**Keywords:** chiral auxiliaries, enzymes, Lewis acids, N-glycosylation, 1,3-oxathiolane sugar and nucleosides, separation of racemic nucleosides, stereoselectivity

## Abstract

Sugar-modified nucleosides have gained considerable attention in the scientific community, either for use as molecular probes or as therapeutic agents. When the methylene group of the ribose ring is replaced with a sulfur atom at the 3’-position, these compounds have proved to be structurally potent nucleoside analogues, and the best example is BCH-189. The majority of methods traditionally involves the chemical modification of nucleoside structures. It requires the creation of artificial sugars, which is accompanied by coupling nucleobases via N-glycosylation. However, over the last three decades, efforts were made for the synthesis of 1,3-oxathiolane nucleosides by selective N-glycosylation of carbohydrate precursors at C-1, and this approach has emerged as a strong alternative that allows simple modification. This review aims to provide a comprehensive overview on the reported methods in the literature to access 1,3-oxathiolane nucleosides. The first focus of this review is the construction of the 1,3-oxathiolane ring from different starting materials. The second focus involves the coupling of the 1,3-oxathiolane ring with different nucleobases in a way that only one isomer is produced in a stereoselective manner via N-glycosylation. An emphasis has been placed on the C–N-glycosidic bond constructed during the formation of the nucleoside analogue. The third focus is on the separation of enantiomers of 1,3-oxathiolane nucleosides via resolution methods. The chemical as well as enzymatic procedures are reviewed and segregated in this review for effective synthesis of 1,3-oxathiolane nucleoside analogues.

## Introduction

Among all the biomolecules in an organism, nucleic acids, namely DNA and RNA, have the unique role of storing the genetic code – the nucleotide sequence that specifies the amino acid sequence of proteins that is essential for life on Earth. These molecules play a significant role in replication, transmission, and transcription of genetic material in life forms [1]. Structural analogues similar to the naturally occurring 2'-deoxynucleosides and ribonucleosides, the DNA and RNA building blocks, respectively, are expected to mimic their counterparts during DNA or RNA synthesis, a biological role that is crucial for cellular reproduction [2]. Most of the drugs that are incorporated in the viral DNA upon phosphorylation in vivo block the DNA polymerase enzyme. However, DNA polymerase recognizes 2’,3’-dideoxynucleosides as substrates, which are incorporated into the growing DNA strand. However, the absence of a 3'-hydroxy group prevents further strand elongation. The anticancer and antiviral activity of 2’,3’-dideoxynucleosides is mainly based on inhibition of DNA synthesis, either through the chain termination process or by competitive inhibition [3–4]. These compounds are the structural analogues of the naturally occurring 2’-deoxynucleosides, the building blocks of DNA.

The World Health Organization (WHO) newsroom announced the primary statistics that HIV and cancer remain a significant global public health issue, having claimed over 47.6 million lives so far [5–6]. The statistics confirm that 1 in 6 deaths happening globally are due to cancer [5]. In 2019, 690,000 people died from HIV-related causes worldwide and by the end of 2019, around 38 million people were living with HIV. From these, 1.7 million people were newly diagnosed [6]. Nucleoside analogues have been in clinical use for almost 50 years and have been the mainstay of treating patients with cancer and viral infections [7–8]. The 2’,3’-dideoxynucleoside analogues, such as AZT (zidovudine), ddI (didanosine), ddC (zalcitabine), and d4T (stavudine), are modified examples of the natural nucleosides with β-ᴅ-configuration in the carbohydrate part. These molecules are known to have a common HIV transcriptase inhibition mechanism, in which cytoplasmic enzymes progressively phosphorylate the analogues to 5'-triphosphates. This then competes with the naturally occurring nucleoside triphosphate substrate to bind to cellular DNA polymerase and viral reverse transcriptase [9]. The effectiveness of nucleoside analogues depends on the ability to replicate naturally occurring nucleosides, interfering with viral as well as cellular enzymes and hampering essential metabolism processes of nucleic acid components. Therefore, it was assumed until recently that effective inhibition of the metabolic enzyme is only possible by ᴅ-nucleoside analogues, which have the stereochemistry of natural nucleosides. This was proved to be untrue when the antiviral activity of 1,3-oxathiolane nucleosides with ʟ-configuration was discovered, and this led to the approval of 3TC (lamivudine, (−)-BCH-189, **1**) as an antiviral drug and, among many others, to the use of FTC (emtricitabine, **2**) and ʟ-FMAU (clevudine). ʟ-Nucleosides can have a comparable and often greater antiviral efficacy than the ᴅ-counterparts, with more favorable toxicological profiles and a greater stability [10]. A variety of nucleoside analogues as possible antiviral agents has appeared, possessing the unusual β-ʟ-configuration. Work has been motivated by the fact that, while retaining strong antiviral and/or antibacterial activity, ʟ-nucleosides are typically endowed with lower host toxicity [11–12]. The antiviral activity and cytotoxicity in MT-4 cells showed that racemic (±)-BCH-189 (**1c**) possesses lower anti-HIV activity (ID_50_ = 0.37–1.31 µM) than AZT (ᴅ-nucleoside, ID_50_ = 0.0048–0.0217 µM). However, (±)-BCH-189 (**1c**) appeared to be a more effective antiviral agent than AZT in PBM cells and U937 cells [13]. The BCH-189 core structure bears two stereocenters, and hence four stereoisomers are possible. The individual stereoisomers were also evaluated against HIV-1 activity in PBM cells and based on this study, it was found that out of four stereoisomers, the β-configured ʟ-(−)-enantiomer **1** (EC_50_ = 0.02 µM) is more potent in primary human lymphocytes than the β-configured ᴅ-(+)-enantiomer **1a** (EC_50_ = 0.2 µM) in CEM cells [14]. Similarly, the 5-fluoro-substituted analogue of cytidine, i.e., β-configured ʟ-(−)-enantiomer **2**, exhibits potential antiviral activity against HIV-1 (EC_50_ = 0.009 µM) in CEM cells. However, the corresponding ᴅ-(+)-enantiomer is less active against HIV-1 (EC_50_ = 0.84 µM) [15]. The fusion of an appropriate sugar element, carbacycle, or heterocyclic equivalent with an activated base results in the corresponding analogues of ᴅ- and ʟ-configured nucleosides and other unnatural nucleoside derivatives [16–19]. Therefore, further demand for various effective chemical syntheses of these nucleoside analogues is rapidly growing.

The FDA has approved modified nucleoside analogues such as zidovudine, didanosine, zalcitabine, stavudine, lamivudine (**1**), and abacavir (a carbanucleoside) for treating HIV infection, along with protease and nonnucleoside reverse transcriptase inhibitors (NNRTIs). Phosphorylation of 1,3-oxathiolane nucleosides, such as 3TC (**1**) and FTC (**2**), occurs in vivo to compete with natural deoxynucleotides for incorporation into (viral) DNA. Chain elongation via reverse transcriptase is thus inhibited. This class of drugs is referred to as nucleoside reverse transcriptase inhibitors (NRTIs). In NRTIs, 3TC (**1**) possesses chemical and biological properties, a sulfur atom instead of C-3', and an unnatural ʟ-configured sugar [20]. The presence of oxygen as a second heteroatom in the sugar ring was also found to result in anti-HIV activity in 1,3-dioxolane nucleosides [21]. A good example for the preparation of 2’,3’-dideoxy-3’-oxacytidine in a stereospecific manner was reported by Chu et al. [22]. Choi et al. [23] produced the 5-fluoro-substituted analogue of a 1,3-oxathiolane nucleoside as a racemic mixture, and the enantiomers were separated using pig liver esterase (PLE) enzyme, which resulted in 5’-butyroyl ester derivatives. They further explained the higher antiviral activity and lower toxicity of the unnatural ʟ-(−)-enantiomer over the ᴅ-(−)-enantiomer. The enantiomers of natural nucleosides are known to have a greater biological activity since they possess structural and configurational similarity to naturally occurring counterparts. In turn, for oxathiolane nucleoside analogues, it was noticed that unnatural (−)-enantiomers have higher anti-HIV activity and lower toxicity in comparison to natural (+)-enantiomers. The activation of such analogues was established to occur preferentially by the enzymes (kinases) or by the target enzymes (polymerases), which may be responsible for such differences [24].

**Figure 1:**
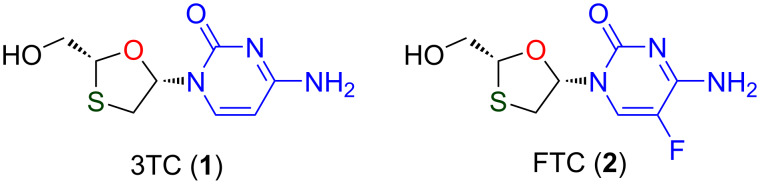
Representative modified 1,3-oxathiolane nucleoside analogues.

Initial results point at a conventional mechanism of action. Therein, the investigation of the cellular metabolism predicts triphosphate formation of the compounds by phosphorylation, and the resulting nucleotide is a selective inhibitor of the HIV-1 reverse transcriptase [25]. Nucleosides with sulfur atom-containing heterocyclic sugar rings at the 3’-position are important pharmaceutical substances. Two well-known important molecules in this category are lamivudine (**1**) and emtricitabine (**2**), as shown in Figure 1.

**Figure 2:**
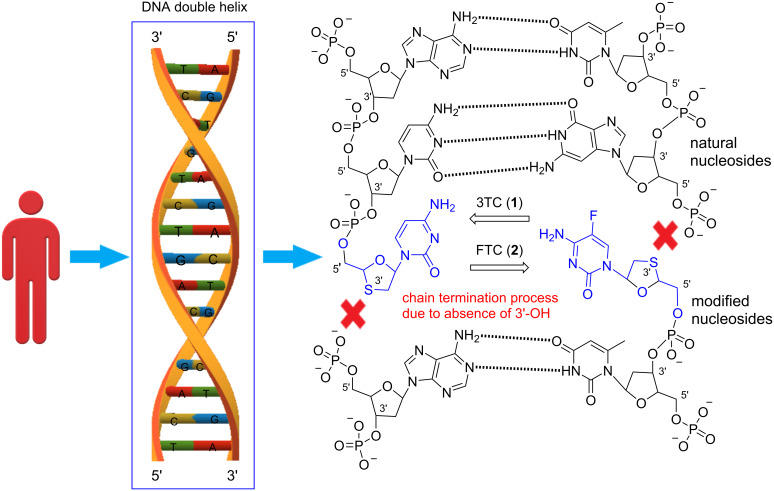
Mechanism of antiviral action of 1,3-oxathiolane nucleosides, 3TC (**1**) and FTC (**2**), as chain terminators.

It was found that there is a remarkable reduction in deaths related to HIV/AIDS in the United States due to usage of combination drug therapies [26]. In these combination therapies, ʟ-(−)-2’,3’-dideoxy-3’-thiacytidine (**1**) is one of the standard components, having an enhanced pharmacological profile over AZT and other dideoxynucleotide inhibitors [26–28]. 3TC (**1**) has a β-ʟ-oxathiolane ring structure, instead of the ribose ring in the canonical nucleosides, and studies have shown that the triphosphate of **1** (i.e., 3TCTP) inhibits reverse transcriptase due to DNA chain termination [26,29–30]. In comparison to some of the other NRTIs that are hardly effective inhibitors of HIV-1 reverse transcriptase, 3TC (**1**) acts as a good substitute with lower toxicity. This could be because it is an unfavorable substrate for mitochondrial DNA polymerases [26–27,30]. The drug triphosphate interferes with HIV reverse transcriptase by competing with natural nucleotides for incorporation into the growing HIV DNA chain. The result if the triphosphate is taken up is the termination of the chain elongation because the drug lacks the 3’-hydroxy group on the deoxyribose ring that is necessary for the sugar–phosphate linking as shown in Figure 2.

The chemical approaches that were broadly used in the past to access these compounds are separated into two main groups: i) those that modify intact nucleosides by modifying the sugar, nucleobase, or both and ii) those that modify the sugar and introduce a nucleobase to a suitable position of the sugar. Since there is more than one chiral center in the structure of these nucleosides, the possibility of stereoisomer formation exists. In most cases, only one stereoisomer is found to be potent and the remaining, undesired isomers are significantly more toxic. Thus, it remains crucial for chemists to establish synthetic approaches toward single desired isomers. The methodologies for modified nucleosides are also known as linear approach and convergent approach [3]. We recognized that there are three major obstacles that have to be cleared: i) efficient preparation of the oxathiolane sugar ring, ii) a stereoselective N-glycosylation process that is compatible with an enantiomerically pure substrate, and iii) separation of enantiomers by chemical or enzymatic resolution methods. This review summarizes the methods used to synthesize 1,3-oxathiolane nucleosides. Many methods provide the formation of a diastereomeric mixture or a racemate of the resultant nucleosides [31]. However, the enantiomers of chiral drugs have indistinguishable chemical and physical properties in an achiral environment. One enantiomer may exhibit a more diverse pharmacological and chemical behavior than the other enantiomer in a chiral environment [32]. Additionally, on the grounds that living systems are, in a sense, themselves chiral, each of the enantiomers of a chiral drug can perform very differently in vivo. Therefore, there is a requirement to synthesize enantiomerically pure nucleosides that are free from undesired isomers.

Over the past three decades, several research groups have been working on devising novel methods for installing glycosidic linkages during the synthesis of modified nucleosides. For 1,3-oxathiolane nucleosides, to achieve β-selective glycosylation, a certain key intermediate was employed in the earlier studies, from 1989 to 2013. The several significant studies have been thoroughly reviewed in 2003 by Chu et al. [33]. The book contains a thorough section on the biological importance and synthesis of oxathiolane nucleosides. Herein, we tried to explore recent developments in comparison to previously reported methods to access 1,3-oxathiolane nucleosides. Similarly, a book chapter by D’alonzo and Guaragna published in 2013 summarizes the synthesis and biological applications of these important analogues [34]. However, a brief account is presented in this section for the sake of continuity.

While targeting to discover antiviral agents [35–37], particularly the class of dideoxynucleotides, it is essential to investigate possible fundamental alteration of the furanose ring and the practical and convenient synthesis of these analogues. These investigations are needed to improve the logic while depicting comparison with the established series of nucleoside analogues. In chiral synthesis, it is often important to establish the ratio of enantiomers before focusing on the isolation of a specific enantiomer. Therefore, having a good overview on enzymatic and chemical resolution methods, for example for the resolution of the oxathiolane nucleoside herein, is beneficial [24].

## Review

### Construction of the 1,3-oxathiolane sugar ring

**Figure 3:**
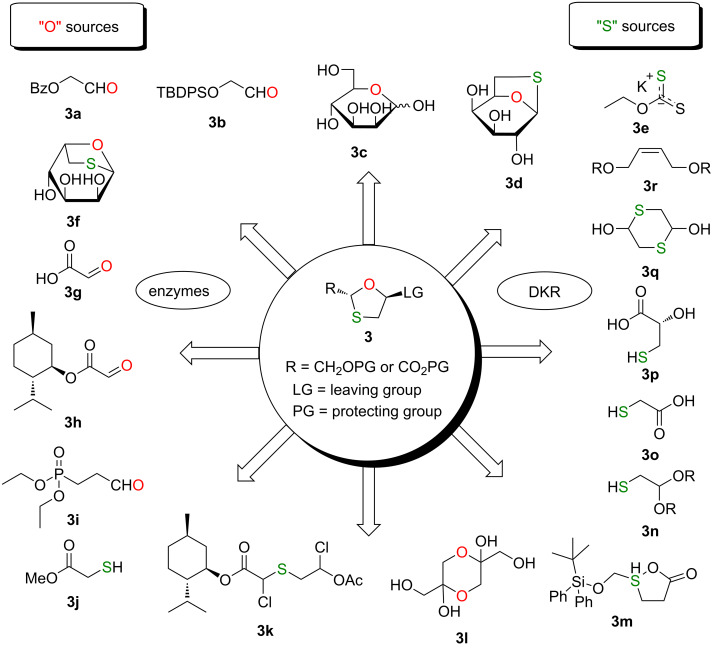
Synthetic strategies for the construction of the 1,3-oxathiolane sugar ring.

The 1,3-oxathiolane ring structure has been known for a long time. However, in recent years, that ring has been utilized in place of the sugar ring in nucleoside analogues. The enantiomerically pure 1,3-oxathiolane core has been an important building block in precursors that result in a defined stereochemistry of the resultant nucleoside product after N-glycosylation. Dynamic kinetic resolution (DKR) is a processes that interconverts a racemic mixture into a single enantiomer via an in situ stereoinversion, and it was implemented in some of the examples described herein. Therefore, for the construction of the 1,3-oxathiolane sugar ring, an extensive number of efficient and environmentally friendly chemical and enzymatic approaches has been established (Figure 3).

### Chemical approaches

Modified sugar rings containing a sulfur heteroatom at C-3' are found in medicinal chemistry. The reaction between oxygen-containing substrates (such as aldehydes or acetals) and sulfur sources (such as thiols or sulfenyl compounds) is one of the most important methods to give the 1,3-oxathiolane sugar ring. Herein, the research on 1,3-oxathiolane sugar ring formation strategies, mainly starting from oxygen- and sulfur-containing substrates, is summarized.

**Scheme 1:**
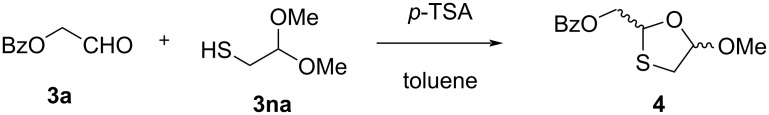
Synthesis of **4** from benzoyloxyacetaldehyde (**3a**) and 2-mercapto-substituted dimethyl acetal **3na**.

In 1989, Belleau and co-workers [38] produced the first oxathiolane nucleoside as a racemic mixture, popularly known as (±)-BCH-189 (**1c**). The key oxathiolane **4**, a precursor of the corresponding nucleoside, was obtained as a 1:1 mixture of anomers (60%) from benzoyloxyacetaldehyde (**3a**) and 2-mercapto-substituted dimethyl acetal **3na**. The reaction was performed in toluene in the presence of *p-*toluenesulfonic acid (*p*-TSA) catalyst at reflux (Scheme 1).

**Scheme 2:**
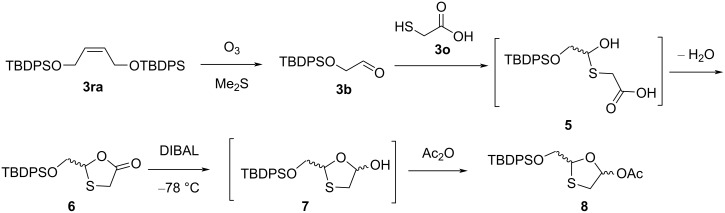
Synthesis of **8** from protected glycolic aldehyde **3b** and 2-mercaptoacetic acid (**3o**).

Sadayoshi and co-workers [39] developed the synthesis of 1,3-oxathiolane derivative **8** (Scheme 2). The protected glycolic aldehyde **3b** was isolated after ozonolysis of alkene **3ra**. The reaction between an aldehyde **3b** and 2-mercaptoacetic acid (**3o**) was carried out at reflux temperature in toluene to synthesize the 1,3-oxathiolane lactone **6** via intermediate **5** after elimination of a water molecule. This was further reduced with diisobutylaluminum hydride (DIBAL) in toluene at −78 °C or by lithium tri*-tert*-butoxyaluminum hydride in THF at 0 °C to obtain lactol **7**, which was subsequently acetylated with acetic anhydride to afford **8** as a 2:1 mixture of anomers.

**Scheme 3:**
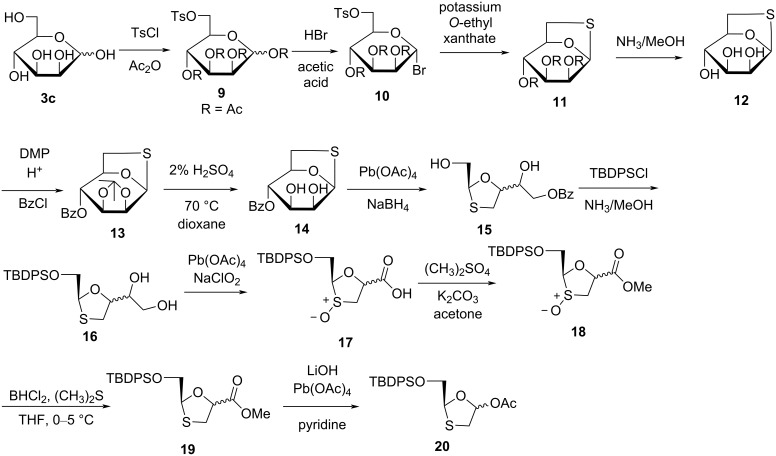
Synthesis of **20** from ᴅ-mannose (**3c**).

Chu and co-workers [40] applied a novel strategy for the synthesis of enantiomerically pure (+)-BCH-189 (**1a**) using ᴅ-mannose (**3c**) as a starting material (Scheme 3). 1,2,3,4-Tetraacetyl-ᴅ-mannose derivative **9** was prepared from ᴅ-mannose (**3c**) by protecting the primary alcohol with a tosyl group, followed by protection of the four hydroxy groups by acetylation. Further, bromo-substituted sugar compound **10** was obtained by a bromination reaction of the anomeric acetyl group. 1,6-Thioanhydro-β-mannose derivative **11** was obtained by cyclization with 3 equivalents of potassium *O*-ethyl xanthate. It was then treated with a methanolic ammonia solution to give triol compound **12**. The protection of the *cis*-2,3-vicinal hydroxy groups of **12** with an isopropylidene, followed by benzoylation, gave compound **13**. Using 2% aqueous sulfuric acid, the isopropylidene group of **13** was selectively deprotected at 70 °C in dioxane to obtain diol **14**. This diol was further cleaved using lead tetraacetate, and further reduction with sodium borohydride produced compound **15**. The 5'-hydroxy group of **15** was then treated with *tert*-butyldiphenylsilyl chloride (TBDPSCl) for selective protection. The compound was further debenzoylated by ammonolysis, which gave compound **16**. Compound **16** underwent oxidative cleavage using lead tetraacetate, and the intermediate aldehyde was oxidized to the carboxylic acid using sodium chlorite, which afforded acid derivative **17**. This was obtained as a mixture of *endo*- and *exo*-sulfoxides. Esterification of **17** was carried out with dimethyl sulfate to give methyl ester **18**, which was further reduced using dichloroborane and dimethyl sulfide to provide sulfide **19** in 80% yield in THF as solvent. Hydrolysis of compound **19** provided the corresponding carboxylic acid, and further oxidative decarboxylation with lead tetraacetate and pyridine provided oxathiolane **20**.

**Scheme 4:**
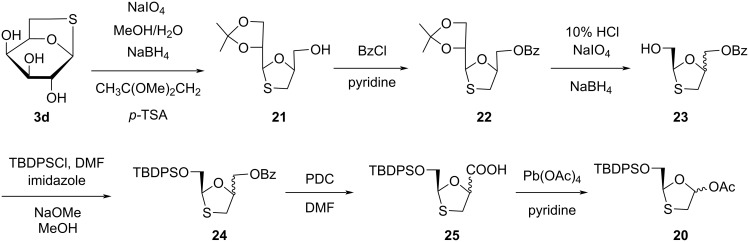
Synthesis of **20** from 1,6-thioanhydro-ᴅ-galactose (**3d**).

Chu and co-workers [41–42] further established a more proficient system for the synthesis of (+)-BCH-189 (**1a**) from 1,6-thioanhydro-ᴅ-galactose (**3d**, Scheme 4). Sodium periodate was used for oxidative cleavage of *cis*-diol **3d**. The subsequent aldehyde was then converted to a vicinal diol by reduction with sodium borohydride. Further, it was protected by 2,2-dimethoxypropane to give the 1,3-oxathiolane derivative **21**. The benzoylated compound **22** was obtained by reaction of benzoyl chloride in pyridine to protect the hydroxy group, which results in a high yield. The isopropylidene group was selectively deprotected using 10% HCl, followed by oxidative breakage of the carbon–carbon bond of the resulting diol using sodium periodate. Further reduction of the aldehyde into a primary alcohol with sodium borohydride affords compound **23**. The protection of the hydroxy group of compound **23** was carried out by TBDPSCl in the presence of imidazole and *N*,*N*-dimethylformamide (DMF) as solvent, and deprotection of the benzoyl group by ammonolysis provides silylated compound **24**. Reaction of **24** with pyridinium dichromate (PDC) in DMF solvent afforded the acid derivative **25**. This derivative was converted to the key intermediate **20** by oxidative decarboxylation [33].

**Scheme 5:**
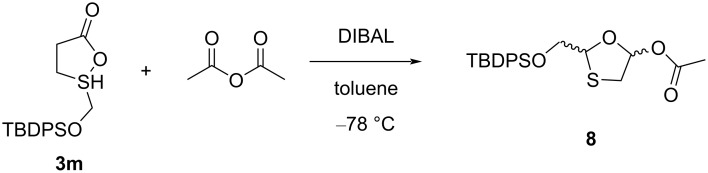
Synthesis of **8** from 2-(*tert*-butyldiphenylsilyloxy)methyl-5-oxo-1,2-oxathiolane (**3m**).

Han et al. [43] developed a method for the novel oxathiolane intermediate 2-(*tert*-butyldiphenylsilyloxy)methyl-5-acetoxy-1,3-oxathiolane (**8**) from 2-(*tert*-butyldiphenylsilyloxy)methyl-5-oxo-1,2-oxathiolane (**3m**, Scheme 5). Compound **3m** was dissolved in toluene and cooled to −78 °C. Further, a DIBAL solution was added slowly while maintaining the reaction temperature below −70 °C. The reaction mixture was further treated with acetic anhydride at room temperature. After workup by adding water and diethyl ether, the reaction mass was filtered and distilled until a residue was obtained. The colorless liquid compound **8** was obtained in 64% yield (as 6:1 mixture of anomers) after flash chromatography with 20% ethyl acetate in hexanes.

**Scheme 6:**
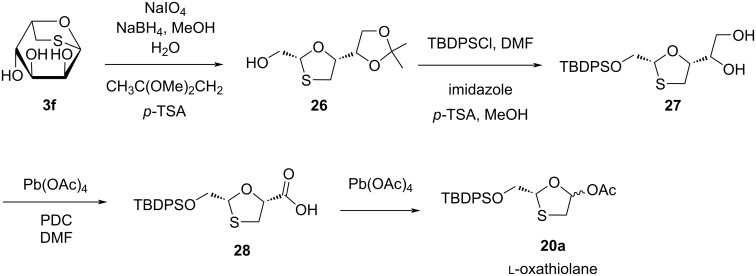
Synthesis of **20a** from ʟ-gulose derivative **3f**.

Chu and colleagues [44] constructed a synthetic approach to access (−)-BCH-189 (**1**) from ʟ-gulose derivative **3f** (Scheme 6). Compound **26** was obtained by oxidation, reduction, and protection of the primary hydroxy group from **3f**. Further, lead tetraacetate directly cleaved diol **27** at room temperature, and oxidation with a mild oxidizing agent, PDC, provided **28**. Using the reaction of lead tetraacetate with **28** via oxidative decarboxylation afforded oxathiolane acetate derivative **20a**.

**Scheme 7:**
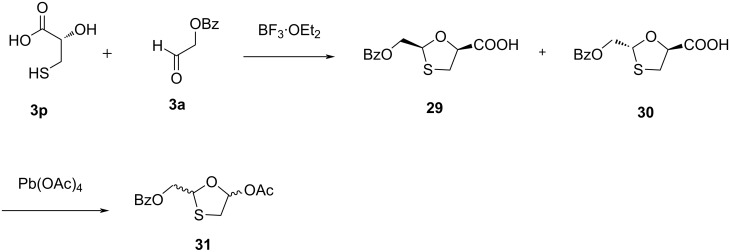
Synthesis of **31** from (+)-thiolactic acid **3p** and 2-benzoyloxyacetaldehyde (**3a**).

The synthesis of a 1,3-oxathiolane precursor required for the preparation of 3TC (**1**) in four steps was reported by Humber et al. [45]. They started with a coupling reaction of (+)-thiolactic acid **3p** and 2-benzoyloxyacetaldehyde (**3a**) using boron trifluoride etherate. A diastereomeric mixture of oxathiolane acids **29** and **30** was prepared in a 1:2 ratio in good yield (Scheme 7). Further separation of the diastereomers by silica gel column chromatography and reaction with lead tetraacetate provided the key oxathiolane derivative **31**.

**Scheme 8:**
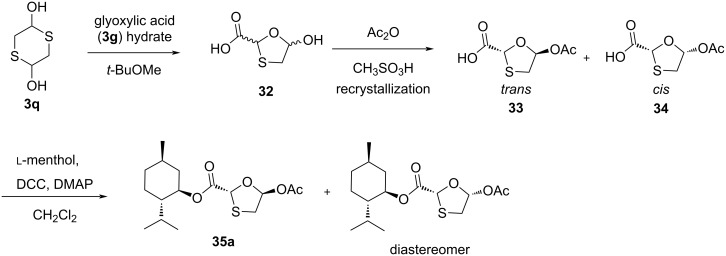
Synthesis of **35a** from 1,4-dithiane-2,5-diol (**3q**) and glyoxylic acid (**3g**) hydrate.

In 1995, Jin et al*.* [46] carried out the reaction of 1,4-dithiane-2,5-diol (**3q**) with glyoxylic acid (**3g**) hydrate at reflux temperature in *tert*-butyl methyl ether, which provided the hydroxyoxathiolane **32**. Further, acetylation of the hydroxyoxathiolane in the presence of methanesulfonic acid gave a 1:2 mixture of the *trans*-diastereomer **33** and the *cis*-diastereomer **34**. The esterification using ʟ-menthol as a chiral auxiliary resulted in a diastereomeric mixture, which was successfully recrystallized to obtain the enantiomerically pure ʟ-menthyl ester **35a** (Scheme 8).

**Scheme 9:**
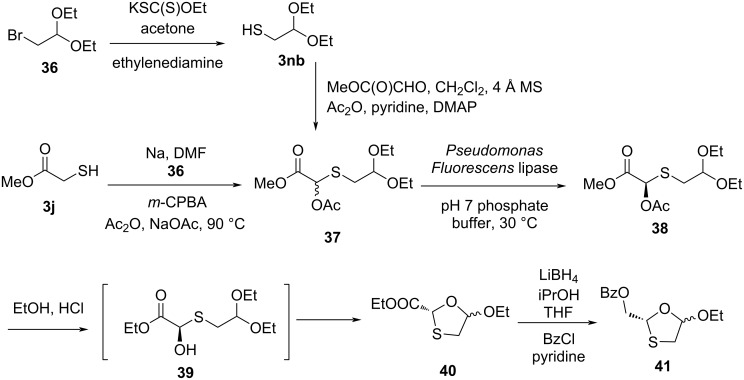
Synthetic routes toward **41** through Pummerer reaction from methyl 2-mercaptoacetate (**3j**) and bromoacetaldehyde diethyl acetal (**36**).

Milton et al. [47] synthesized the key intermediate **38** by two synthetic routes. The first route involves a reaction of bromoacetaldehyde diethyl acetal (**36**) with a xanthate ester, followed by treatment of ethylenediamine, which afforded the thiol compound **3nb**. Further treatment of the thiol **3nb** with methyl glyoxylate in dichloromethane solvent along with molecular sieves (4 Å), followed by in situ acetylation using Ac_2_O, pyridine, and catalytic 4-(*N*,*N*-dimethylamino)pyridine (DMAP) provided compound **37**. The second route involves condensation of the sodium salt of methyl 2-mercaptoacetate (**3j**) with bromoacetaldehyde diethyl acetal (**36**) in DMF solvent and further oxidation of the sulfide using *m*-CPBA, followed by Pummerer rearrangement using Ac_2_O and sodium acetate at 90 °C, which provides compound **37** (Scheme 9). α-Acetoxy sulfide intermediate **37** was resolved using a lipase in *t-*BuOMe, resulting in a high enantiomeric excess. They used an enzymatic resolution of an acetoxy sulfide with a *Pseudomonas fluorescens* lipase to obtain compound **38**. Reaction of chiral acetoxy sulfide **38** with HCl in dry ethanol induced acetate removal by transesterification to give the hemithioacetal **39**, which cyclized to the oxathiolane **40** in situ with minor isomerization. The reduction of the ester group with LiAlH_4_, followed by benzoylation using benzoyl chloride and pyridine gave 1,3-oxathiolane derivative **41**.

**Scheme 10:**
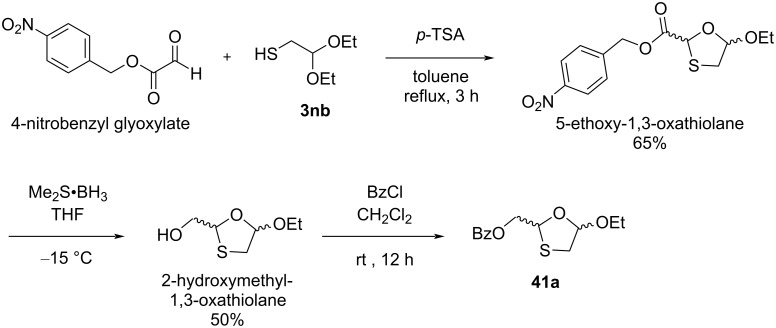
Strategy for the synthesis of 2,5-substituted 1,3-oxathiolane **41a** using 4-nitrobenzyl glyoxylate and mercaptoacetaldehyde diethyl acetal (**3nb**).

Kraus and Attardo [48] developed new strategies for the synthesis of a new 2,5-substituted 1,3-oxathiolane intermediate (Scheme 10). The approach involved the cyclocondensation reaction of anhydrous 4-nitrobenzyl glyoxylate with mercaptoacetaldehyde diethyl acetal (**3nb**) at reflux temperature in toluene solvent. This led to the formation of a 5-ethoxy-1,3-oxathiolane derivative. Further, reduction of the ester functionality with borane dimethyl sulfide at −15 °C afforded the corresponding 2-(hydroxymethyl)-1,3-oxathiolane in 50% yield. It was further treated with benzoyl chloride in the presence of triethylamine (TEA), which provided the desired compound **41a** as 1:1 mixture of *cis*- and *trans*-isomers.

**Scheme 11:**
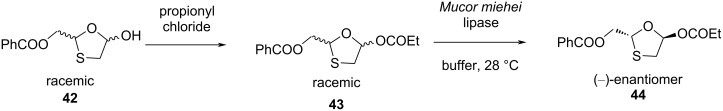
Synthesis of **44** by a resolution method using *Mucor miehei* lipase.

In 1995, Cousins and co-workers [49] investigated enzymatic methods to resolve an oxathiolane intermediate (Scheme 11). Racemic intermediate **42** was converted into **43** with propionyl chloride protection. The procedure provides the enzymatic resolution of oxathiolane propionate derivative **43** by using *Mucor miehei* lipase, which affords (−)-enantiomer **44** as residual substrate. This enantioenriched precursor was useful to obtain the pure corresponding nucleoside analogue.

**Scheme 12:**
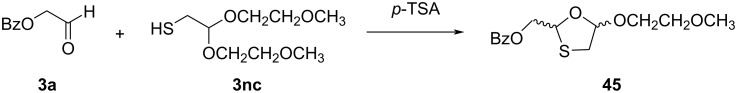
Synthesis of **45** from benzoyloxyacetaldehyde (**3a**) and 2-mercaptoacetaldehyde bis(2-methoxyethyl) acetal (**3nc**).

Faury and co-workers [50] synthesized the tetrazole analogues of 1,3-oxathiolane nucleosides to show the antiviral activity in comparison to ribavirin. The condensation reaction between benzoyloxyacetaldehyde (**3a**) and 2-mercaptoacetaldehyde bis(2-methoxyethyl) acetal (**3nc**) in the presence of *p-*TSA as catalyst afforded the intermediate 2-benzoyloxymethyl-5-(2-methoxyethyloxy)-1,3-oxathiolane **45** (Scheme 12).

**Scheme 13:**
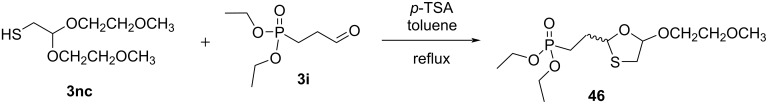
Synthesis of **46** from 2-mercaptoacetaldehyde bis(2-methoxyethyl) acetal (**3nc**) and diethyl 3-phosphonoaldehyde **3i**.

Kraus [51] developed the cyclocondensation of 2-mercaptoacetaldehyde bis(2-methoxyethyl) acetal (**3nc**) with diethyl 3-phosphonoaldehyde (**3i**) to provide the novel oxathiolane intermediate **46** (Scheme 13). The reaction was carried out in the presence of *p*-TSA at reflux temperature in toluene solvent.

**Scheme 14:**
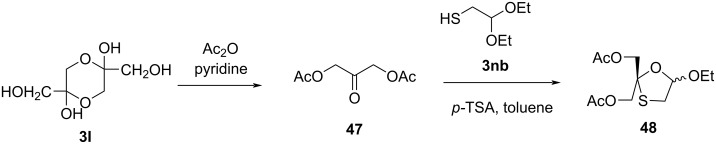
Synthesis of **48** from 1,3-dihydroxyacetone dimer **3l**.

The synthesis and antiviral evaluation of 4'-(hydroxymethyl)oxathiolane nucleosides was reported by Chao and Nair [52]. The synthetic approach used 1,3-dihydroxyacetone dimer **3l** (Scheme 14). This dimer, upon acetylation using acetic anhydride in pyridine, produced compound **47**. Further, cyclocondensation of **47** with 2-mercaptoacetaldehyde diethyl acetal (**3nb**) in the presence of *p*-TSA in benzene solvent afforded 1,3-oxathiolane intermediate (±)-2,2-bis(acetoxymethyl)-5-ethoxy-1,3-thioxalane (**48**).

**Scheme 15:**
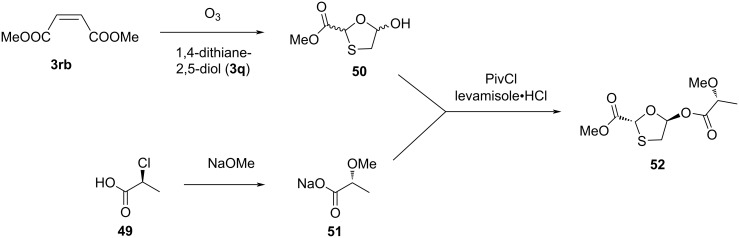
Approach toward **52** from protected alkene **3rb** and lactic acid derivative **51** developed by Snead et al.

More recently, an approach developed by Snead et al. [53] at the Medicines for All Institute used lactic acid derivatives to test the impact of a chiral auxiliary on N-glycosylation. Compound **50** was synthesized by ozonolysis of alkene **3rb**, followed by reaction of aldehyde (generated in situ from alkene) with 1,4-dithiane-2,5-diol (**3q**). The use of lactic acid derivatives provided both enantiomers of oxathiolane precursors. The use of an (*S*)-lactic acid derivative resulted in the formation of an oxathiolane precursor with the opposite configuration of the desired one, which eventually led to the opposite enantiomer of 3TC (**1a**). Therefore, the authors changed the procedure and used the (*R*)-lactic acid derivative **51** to facilitate the formation of 3TC (**1**). In this procedure, the oxathiolane **50** was acylated using the lactic acid derivative sodium (*R*)-2-methoxypropanoate (**51**), which provided the derivative **52** (Scheme 15). Compound **51** was obtained by reaction of (*S*)-2-chloropropanoic acid (**49**) with sodium methoxide. Further, selective recrystallization in an appropriate solvent (toluene/hexanes) resulted in a single isomer (50:1 dr) in solution. The oxathiolane derivative **52** has the opposite configuration of that required for 3TC (**1**) synthesis. This acylation reaction was accomplished using pivaloyl chloride in the presence of levamisole, which gave an improved overall yield of **52** of up to 67%. In this approach, the required stereochemistry of the thioacetal compound was created, so that the coupling with a nucleobase in a further step determines the stereochemistry of the attaching nucleobase at the anomeric center, which is governed by an anchimeric effect. Thus, the method determines the configuration of proximal as well as remote stereocenters in a single step, and both enantiomers of the β-nucleoside were accessed from affordable starting materials.

**Scheme 16:**
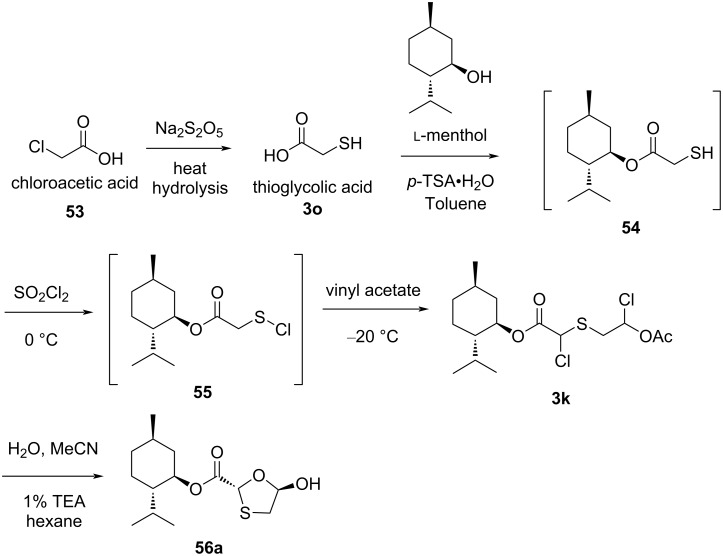
Recent approach toward **56a** developed by Kashinath et al.

Kashinath and co-workers [54] also identified an innovative route to access an oxathiolane intermediate, which was further used for the synthesis of lamivudine (**1**) as well as emtricitabine (**2**). They presented an efficient reaction path that utilized commonly available and inexpensive starting materials. Sulfenyl chloride chemistry was used to synthesize the oxathiolane precursor **56a** from acyclic precursors. The method used chloroacetic acid (**53**), vinyl acetate, sodium thiosulfate, and water to construct the oxathiolane moiety. The use of sulfenyl chloride provided a new method to access such oxathiolanes (Scheme 16). Thioglycolic acid (**3o**), upon reaction with ʟ-menthol, afforded the relevant thiol-substituted esters **54**, which further reacted with sulfuryl chloride to give compound **55**. The reaction of compound **55** with vinyl acetate constructed a sulfur–carbon bond and produced **3k**. The sulfuryl chloride reagent simultaneously allowed for chlorination at the α-position of the ester. The dichloro-substituted intermediate **3k** was further cyclized to produce the oxathiolane **56a** by reaction with water in the presence of acetonitrile as solvent. The focus of this novel route was to access basic reagents that are useful for the synthesis of 3TC (**1**) and FTC (**2**).

**Scheme 17:**
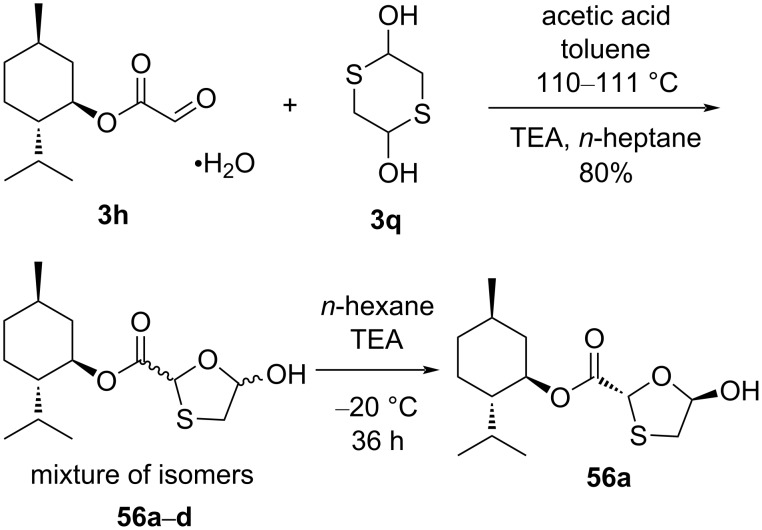
Synthesis of **56a** from ʟ-menthyl glyoxylate (**3h**) hydrate by DKR.

One of the methods of choice for the industrial manufacturing of lamivudine (**1**) follows the procedure suggested by Whitehead et al. [55]. This procedure involves the use of compound **56a**, where an ʟ-menthyl moiety as chiral auxiliary is connected to an enantiomerically pure oxathiolane-based lactol. This is a necessary requirement to produce the desired stereochemistry in the product. It was extensively reported that the β-selectivity could be due to the formation of an oxonium ion, which is stabilized through anchimeric assistance of the ʟ-menthyl ester function. The method requires highly effective crystallization-induced DKR to achieve an efficient synthesis of enantiomerically pure oxathiolane-based lactol **56a** from ʟ-menthyl glyoxylate (**3h**) monohydrate and 1,4-dithiane-2,5-diol (**3q**, Scheme 17).

**Scheme 18:**
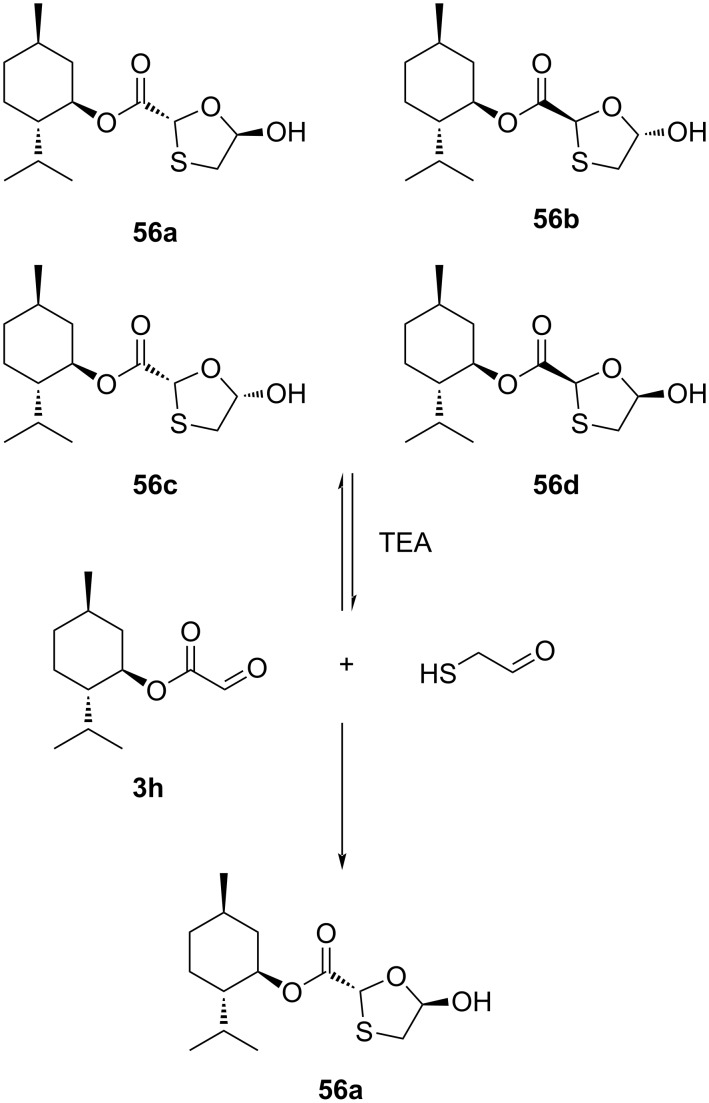
Possible mechanism with catalytic TEA for rapid interconversion of isomers.

The investigation proved that the base TEA was capable of effecting the equilibration at C-2 but advantageous for the crystallization process. A number of bases was also evaluated by this research group: pyridine gave only a small amount of interconversion, whereas TEA caused rapid interconversion. Furthermore, it was discovered that instant interconversion and crystallization of **56a** in 80% yield (Scheme 18) was possible through a mechanism that required the addition of a catalytic amount of TEA.

**Scheme 19:**
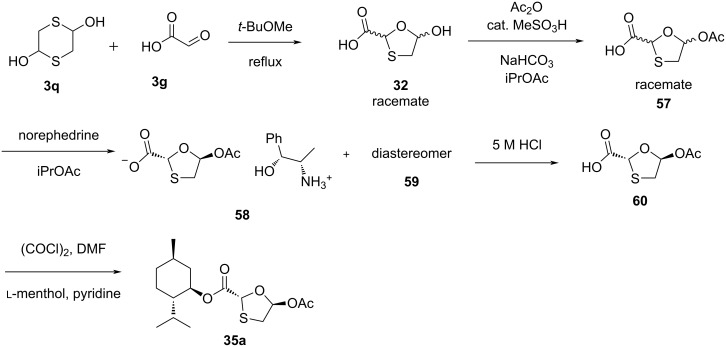
Synthesis of **35a** by a classical resolution method through norephedrine salt **58** formation.

A method was established utilizing a Vorbrüggen reaction [55–56] of 5-acetoxyoxathiolane **35a**, which is an enantiomerically pure compound that can be used for the synthesis of lamivudine (**1**), as summarized in the next section. Access to crystalline 5-acetoxyoxathiolane **35a** was accomplished either by selective crystallization in the presence of the remaining diastereoisomers, although in only 16% yield, or by a classical resolution method using the norephedrine salt **58** (Scheme 19). The other diastereomer **59** remained dissolved in the mother liquor. The treatment of the norephedrine salt **58** with 5 M HCl afforded the enantiopure acid **60**, which was further converted to the desired 1,3-oxathiolane-substituted ʟ-menthyl ester **35a**.

**Scheme 20:**
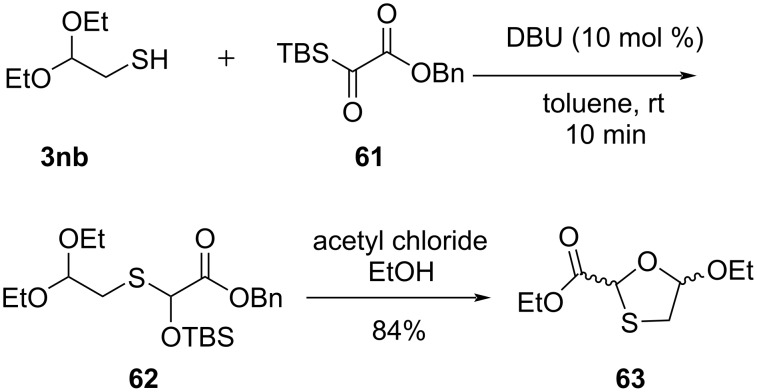
Synthesis of **63** via [1,2]-Brook rearrangement from silyl glyoxylate **61** and thiol **3nb**.

The synthetic use of [1,2]-Brook rearrangement for the synthesis of lamivudine (**1**) and the opposite enantiomer **1a** was demonstrated by Han et al. [57]. They carried out the [1,2]-Brook rearrangement of silyl glyoxylate **61** using thiol **3nb** as the nucleophile. Under optimized conditions, the reaction of the key intermediate **62** with acetyl chloride in ethanol results in the formation of the 1,3-oxathiolane species **63** (Scheme 20).

### Enzymatic approaches

1,3-Oxathiolanes have shown broad biological activities, including the most important intermediates in the synthesis of the pharmaceuticals lamivudine (**1**) and emtricitabine (**2**), which have been approved as drugs to treat HIV infection [58] as well as human chronic hepatitis B [59]. Using asymmetric synthesis or resolution with appropriate enzymes to prepare these enantiopure 1,3-oxathiolanes has gained extensive attention due to the good stereoselectivity, high efficiency, mild reaction conditions, and eco-friendliness.

**Scheme 21:**
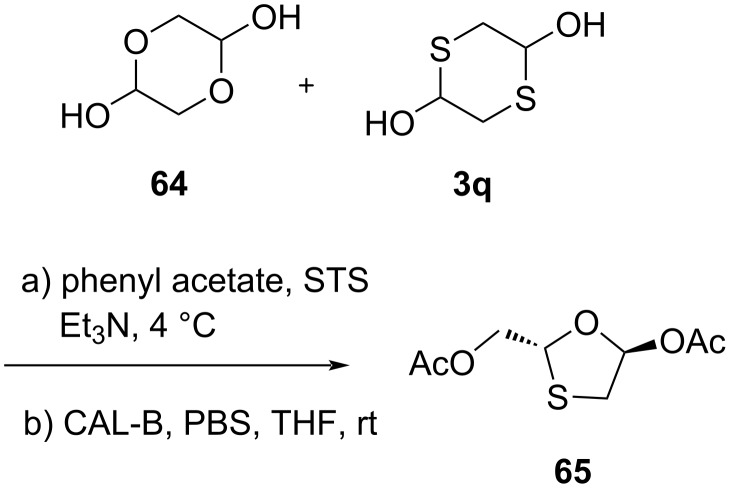
Combined use of STS and CAL-B as catalysts to synthesize an enantiopure oxathiolane precursor **65**.

Ren and colleagues [59] recently reported the preparation of an enantiopure 1,3-oxathiolane **65** utilizing a multienzymatic cascade protocol (Scheme 21). The combined use of surfactant-treated Subtilisin Carlsberg (STS) and *Candida antarctica* lipase B (CAL-B) resulted in the 1,3-oxathiolane ring in THF and phosphate-buffered saline (PBS). The reaction used **64** and **3q** as starting materials and was stereocontrolled efficiently, providing an enantiomeric excess of about >99%. The subsequent N-glycosylation further provided enantiopure lamivudine (**1**). Hu et al. [60] explained that chiral HPLC and nuclear Overhauser effect (NOE) NMR spectroscopy are useful tools to monitor and control the chirality when utilizing a modified 1,3-oxathiolane intermediate **65** obtained via enzyme-catalyzed selective hydrolysis.

**Scheme 22:**
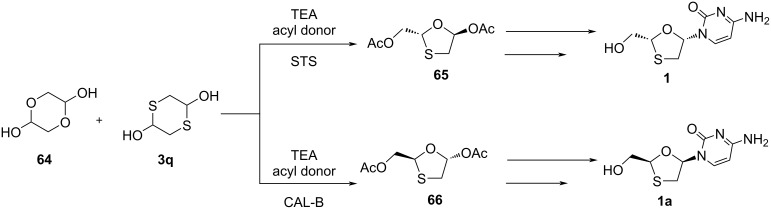
Synthesis of **1** and **1a** from glycolaldehyde dimer **64** and 1,4-dithiane-2,5-diol (**3q**) using STS and CAL-B, respectively.

Hu et al*.* [61] established a green catalyst, STS, for the asymmetric synthesis of lamivudine (**1**). Specifically, this approach used enzyme optimization techniques to efficiently synthesize highly enantiopure nucleoside analogues. The group found that the stereochemistry of the target molecules was selectively obtained using different enzymes. Importantly, the stereochemistry of the 1,3-oxathiolane intermediates **65** and **66** could be controlled well (Scheme 22). The glycolaldehyde dimer **64** and 1,4-dithiane-2,5-diol (**3q**) were reacted in the presence of TEA and the acyl donor phenyl acetate. The presence of CAL-B allowed the formation of the intermediate **66** and ultimately the corresponding nucleoside **1a** in a protocol by Vorbrüggen et al. In turn, using STS, this valuable asymmetric synthesis provided the intermediate **65**, which led to lamivudine (**1**).

**Scheme 23:**
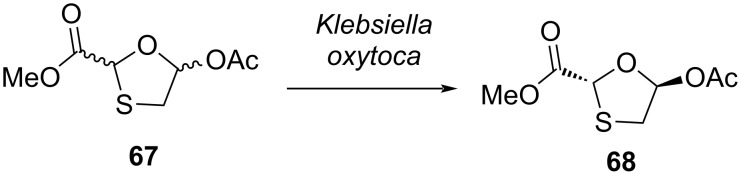
Synthesis of **68** by using *Klebsiella oxytoca.*

Recently, Chen at al. [62] reported the isolation of the strain *Klebsiella oxytoca* from soil by a target-oriented process, and it was utilized as a catalyst for the asymmetric preparation of a chiral intermediate of the antiviral agent lamivudine (**1**, Scheme 23). Further, the reaction conditions were optimized, and a series of factors was explored, including pH value, concentration, temperature, as well as the presence of metal ions and surfactant. Exceptionally, the end product was obtained in 99.9% ee by using whole-cell *Klebsiella oxytoca* catalysis and enantioselective resolution of the racemic mixture at 30 °C, pH 7.0, a substrate concentration of 1.5 g/L, and no additives. As compared to nearly all of the lipase-catalyzed methods to produce the chiral oxathiolane precursor **68** of lamivudine (**1**) from a mixture of isomers, i.e., **67**, the reaction occurred in a single-phase aqueous system, which may be considered a green chemistry approach.

**Scheme 24:**
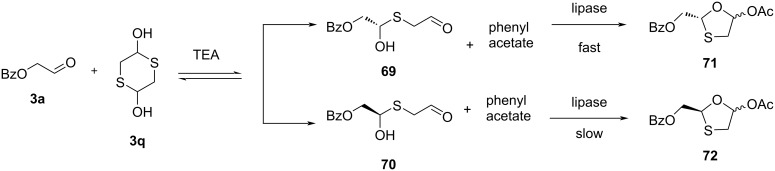
Synthesis of **71** and **72** using *Trichosporon taibachii* lipase and kinetic resolution.

Recently, Zhang and co-workers [63] developed a one-pot enzymatic synthesis of enantiopure 1,3-oxathiolane with *Trichosporon laibachii* lipase and a kinetic resolution. The synthesis of enantiopure ((*R*)-5-acetoxy-1,3-oxathiolan-2-yl)methyl benzoate (**71**) was carried out from the substrates **3a**, 1,4-dithiane-2,5-diol (**3q**), and phenyl acetate via dynamic covalent kinetic resolution. This was a one-pot process that reached 96.5% ee through the combination of the reversible hemithioacetal transformation and the enantioselective lactonization catalyzed by the immobilized lipase from *Trichosporon laibachii* (Scheme 24). As a result, the desired stereochemistry of 1,3-oxathiolane precursors **71** and **72** was achieved.

**Scheme 25:**
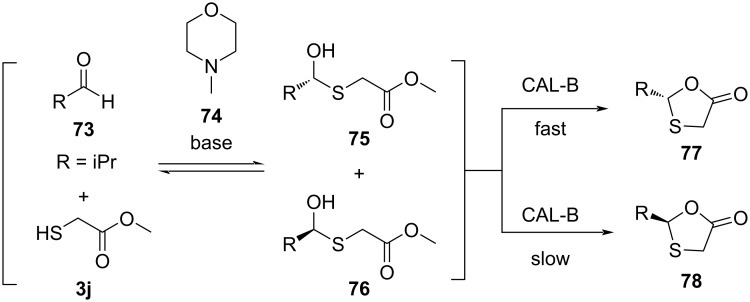
Synthesis of 1,3-oxathiolan-5-ones **77** and **78** via dynamic covalent kinetic resolution.

In 2014, Zhang et al. [64] reported an optimized asymmetric synthesis of 1,3-oxathiolan-5-ones **77** and **78** via dynamic covalent kinetic resolution using hemithioacetal chemistry coupled with a lipase-catalyzed cyclization (Scheme 25). Methyl thioglycolate (**3j**) was used in the reaction with aldehyde **73**. These acted as hemithioacetal substrate and acyl donor, respectively. CAL-B was further utilized for the subsequent intramolecular cyclization of hemithioacetal intermediates **75** and **76**. Screening of base additives showed that good results could be obtained by addition of 4-methylmorpholine (**74**). Enantioselectivity for a wide range of substrates was achieved in good yield with rigorous optimization of the reaction conditions by utilization of wild-type CAL-B.

### Synthetic N-glycosylation strategies for glycosidic C–N bond formation in 1,3-oxathiolane nucleosides

This section will discuss the methods for constructing glycosidic C–N bonds in 1,3-oxathiolane nucleosides. The chemistry detailed in this section will concentrate on building N-nucleosides. There have been several excellent reviews on the construction of nucleosides over the past decades [33–34,65]. Accordingly, this section begins with an introduction on important classical approaches and older yet creative methods to provide the reader with a historical context. For comparison, this will be followed by a discussion of more modern techniques, including chiral auxiliaries for neighboring group participation and transition metal-catalyzed reactions, along with recent new promoter-dependent advances. It is generally agreed that the stereochemical outcome of glycosylation can be affected by multiple factors [66–69], which include i) structure and conformation of the glycosyl substrates, ii) glycosylation reagents or promoters, iii) the solvent, iv) presence of a participating or chiral auxiliary protecting group, v) the presence of a conformationally locked protecting group, vi) the presence of a glycosyl acceptor tethering group, and/or vii) the presence of an exogenous nucleophilic additive.

**Figure 4:**
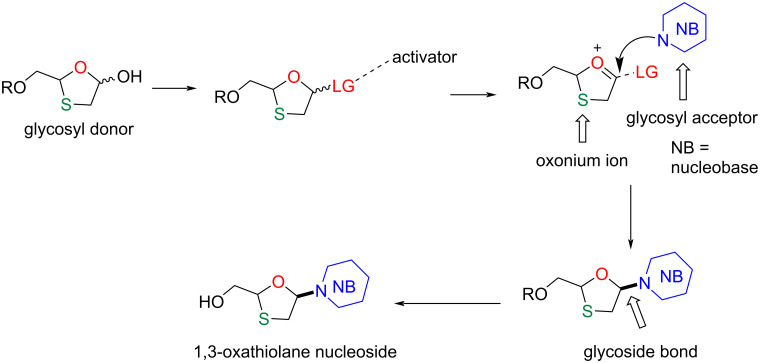
Pathway for glycosidic bond formation.

The distinction between α- and β-glycosidic bonds depends on the relative orientation of the anomeric carbon atom and the stereocenter furthest from position C-1 in the sugar. For example, when the nucleobase at C-1 is oriented *cis* to the hydroxymethyl group of the sugar at C-4, it is a β-glycosidic bond, whereas if it is orientated *trans*, it is referred to as α-glycosidic bond [70]. The exact character of the glycosidic bond in the structure defines the physicochemical properties and biological role of the molecule [71]. There have been numerous efforts to synthesize these nucleoside analogues in order to achieve the desired stereoselectivity during β-selective glycosidic bond formation. The general pathway for glycosidic bond formation (Figure 4) shows that the glycoside donor moiety has to be activated using an appropriate activator to form an oxonium ion. The attack of a nucleobase (glycosyl acceptor) may occur on either side of the oxonium ion, which can result in two anomers, i.e., an α- and a β-anomer. The factors affecting such stereocontrolled glycoside bond formations are also discussed in this review.

**Scheme 26:**
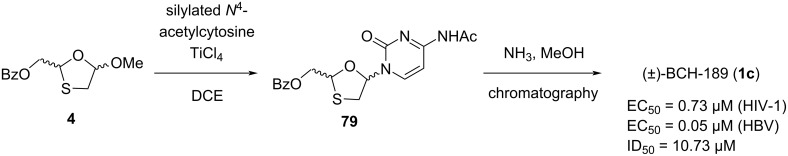
First synthesis of (±)-BCH-189 (**1c**) by Belleau et al.

The preparation of the racemate **1c** was reported by Belleau et al. in 1989 (Scheme 26) [38]. The method involved the coupling of oxathiolane derivative **4** with silylated cytosine, which afforded **79** as a mixture of the *cis*- and *trans*-nucleosides. The process used N-protected cytosine and further chromatographic separation. The deprotection with a methanolic ammonia solution provided racemic (±)-BCH-189 (**1c**). In vitro studies of (±)-BCH-189 (**1c**) showed potent anti-HIV-1 activity. The EC_50_ value of (±)-BCH-189 (**1c**) was reported to be in the range of 0.37–1.31 µM (mean 0.73 µM), and the compound was effective against HIV-1 in MT-4 cells [13].

**Scheme 27:**
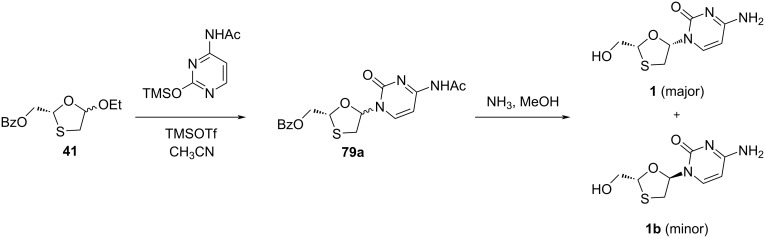
Enantioselective synthesis of 3TC (**1**).

Enantioselective enzymatic synthesis of 3TC (**1**) was also reported by Milton et al. [47], who isolated oxathiolane precursor **41**, as discussed earlier, by enzymatic resolution of an acetoxy sulfide by a *Pseudomonas fluorescens* lipase. Using this pure precursor **41**, the synthesis of 3TC (**1**) was accomplished by N-glycosylation with silylated base using trimethylsilyl trifluoromethanesulfonate (TMSOTf) catalyst to obtain nucleoside derivative **79a**, followed by deprotection using methanolic ammonia (Scheme 27).

**Scheme 28:**
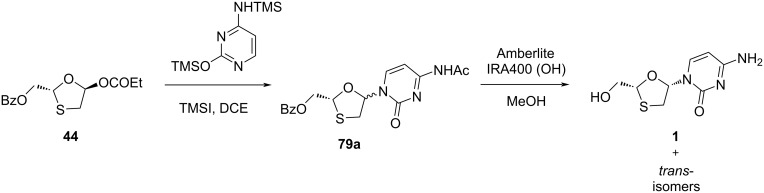
Synthesis of *cis-*diastereomer 3TC (**1**) from oxathiolane propionate **44**.

Cousins et al. [49] carried out the coupling of enantiomerically enriched oxathiolane propionate **44** with silylated cytosine in the presence of the Lewis acid trimethylsilyl iodide (TMSI), which gave a *cis*/*trans* ratio of 1.3:1 for the nucleoside intermediate **79a**. Further, the nucleoside intermediate **79a** was deprotected using a type of basic resin. This gave the *cis*-diastereomer 3TC (**1**), which was purified by chiral HPLC, resulting in an ee value of 70% (Scheme 28).

**Scheme 29:**
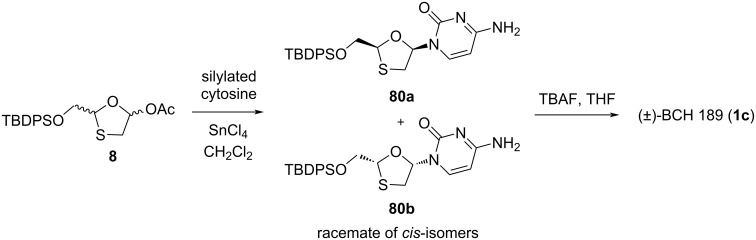
Synthesis of (±)-BCH-189 (**1c**) via SnCl_4_-mediated N-glycosylation of **8**.

Further developments in the effective enantiopure synthesis of lamivudine (**1**) were achieved by many scientists. The synthesis of 1,3-oxathiolane nucleosides utilizing stereoselective coupling of a nucleobase with the oxathiolane sugar intermediate via in situ chelation was reported by Liotta and co-workers (Scheme 29) [72]. Appropriate Lewis acids form a complex with the oxathiolane intermediates via in situ chelation. The exclusive formation of the β-anomer was observed upon coupling of the anomer mixture **8** with silylated cytosine using stannic chloride (about 2 equiv in CH_2_Cl_2_ at room temperature). This stereoselective outcome could have been due to an in situ chelation process. The level of selectivity was determined by HPLC to be >300:1 in favor of the β-configured *cis*-isomers (racemic mixture of **80a** and **80b**) [30]. Further, the desilylation using tetrabutylammonium fluoride (TBAF) gave racemic (±)-BCH-189 (**1c**).

**Scheme 30:**
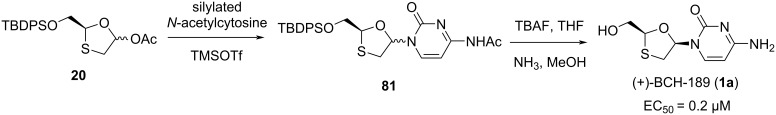
Synthesis of (+)-BCH-189 (**1a**) via TMSOTf-mediated N-glycosylation of **20**.

Chu et al. [40] described coupling of crude 1,3-oxathiolane precursor **20** with silylated acetylcytosine utilizing TMSOTf as a Lewis acid, which gave a mixture of α- and β-anomers (1:2 ratio) of **81** (Scheme 30). The mixture of anomers was further separated by silica gel column chromatography. (+)-BCH-189 (**1a**) and the α-anomer were produced individually by further deacetylation using methanolic ammonia and desilylation with TBAF [33]. (+)-BCH-189 (**1a**) was found to be less active against HIV-1 (EC_50_ = 0.2 µM in CEM cells) than (−)-BCH-189 (**1**, EC_50_ = 0.07 µM in CEM cells) [14].

**Scheme 31:**
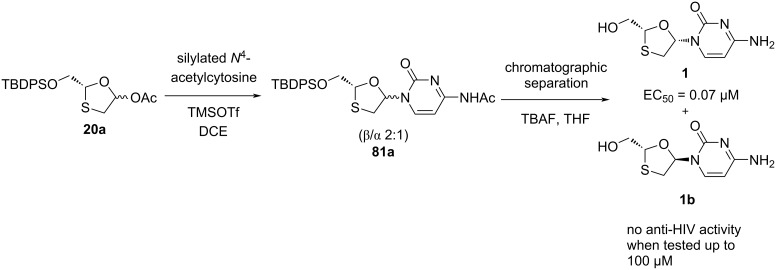
Synthesis of 3TC (**1**) from oxathiolane precursor **20a**.

Chu and co-workers [44] reported a synthetic procedure to access (−)-BCH-189 (**1**). Compound **20a** was synthesized from ʟ-gulose derivative **3f** (Scheme 6). The glycosylation reaction of oxathiolane intermediate **20a** with silylated *N*^4^-acetylcytosine in dichloroethane using TMSOTf as a catalyst gave **81a** as a β/α 2:1 mixture (Scheme 31). Separation by chromatography and deprotection with TBAF in THF afforded the (−)-isomer 3TC (**1**, EC50 = 0.07 µM in CEM cells) and the *trans*-isomer **1b**. The *trans*-substituted (+)-isomer **1b** did not shown any activity when it was tested (up to 100 µM). Further investigation showed that using stannic chloride instead of TMSOTf for the N-glycosylation procedure afforded a racemic mixture. This could be due to the opening as well as closing of the oxathiolane ring under the reaction conditions.

**Scheme 32:**
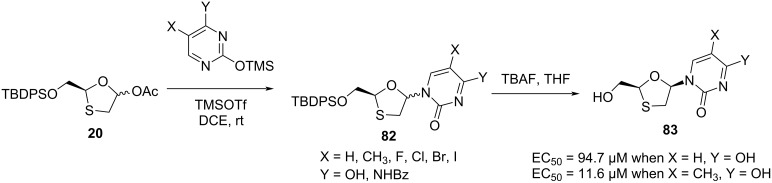
Synthesis of **83** via N-glycosylation of **20** with pyrimidine bases.

**Scheme 33:**
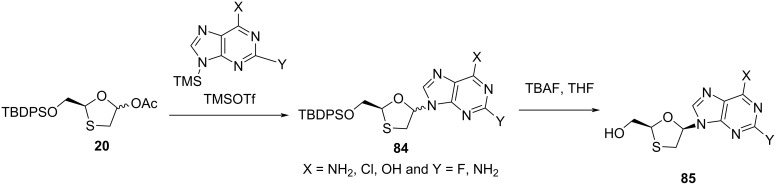
Synthesis of **85** via N-glycosylation of **20** with purine bases.

Optically pure β-ᴅ- and α-ᴅ-configured 1,3-oxathiolane pyrimidine and 1,3-oxathiolane purine nucleosides with natural nucleoside configuration were synthesized by Jeong et al. (Scheme 32 and Scheme 33) [41–42]. The purpose of this was the investigation of the structure–activity relationships as anti-HIV-1 agents. The oxathiolane intermediate **20**, produced from ᴅ-mannitol, was further condensed with a range of pyrimidine and purine nucleobases via N-glycosylation. The anti-HIV activity of the nucleosides **83** was quantified by EC_50_ values of 94.7 µM and 11.6 µM when X = H or CH_3_ and Y = OH, respectively [33]. The α-anomers were also isolated by chromatographic separation methods.

**Scheme 34:**
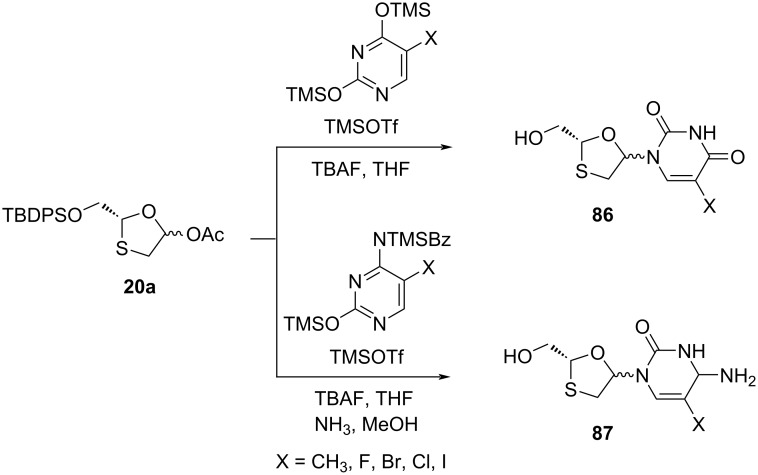
Synthesis of **86** and **87** via N-glycosylation using TMSOTf and pyrimidines.

**Scheme 35:**
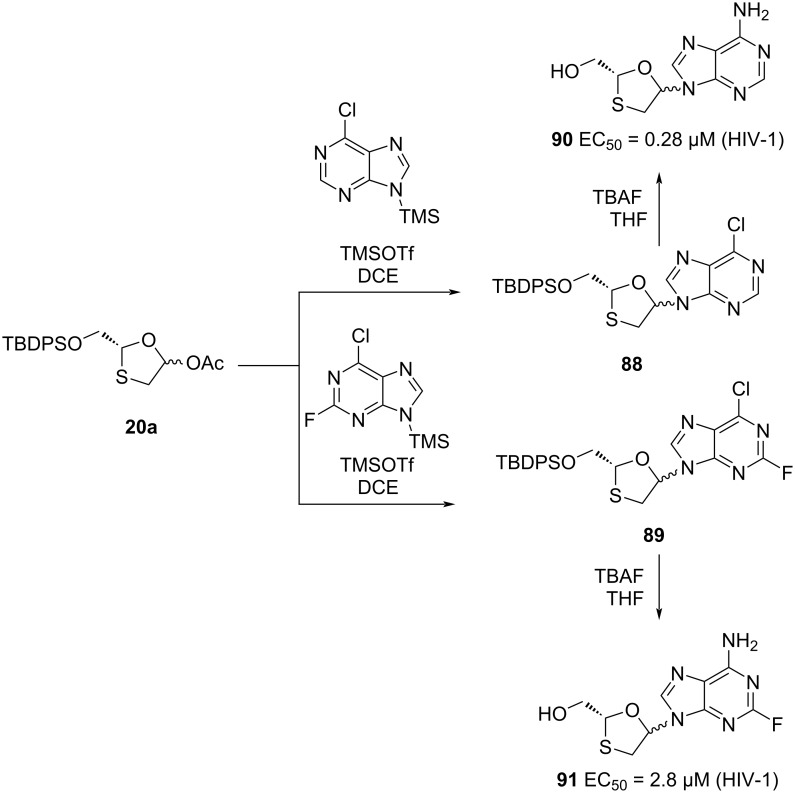
Synthesis of **90** and **91** via N-glycosylation using TMSOTf and purines.

To study the structure–activity relationships of various nucleobase derivatives, oxathiolane acetate **20a** was further condensed with various pyrimidines (Scheme 34) and purines (Scheme 35), as reported by Jeong et al. [73]. When X = F, the cytosine derivative **87**, among all of these nucleosides having anti-HIV activity, was found to be the most potent. The pyrimidine analogues **90** and **91** were also found to be active against HIV-1, with EC_50_ = 0.28 µM and 2.8 µM, respectively [33].

**Scheme 36:**
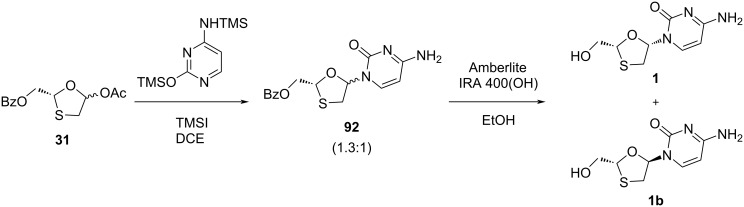
Synthesis of 3TC (**1**) via TMSI-mediated N-glycosylation.

In 1992, Humber et al. [45] established a method for glycosylation of benzoylated oxathiolane **31** with silylated cytosine in the presence of trimethylsilyl iodide (TMSI) as a catalyst, which afforded nucleoside **92** as a β/α 1.3:1 mixture. Furthermore, anomeric mixture separation and deprotection using Amberlite IRA 400(OH) afforded 3TC (**1**) and the α-anomer **1b** (Scheme 36).

**Scheme 37:**
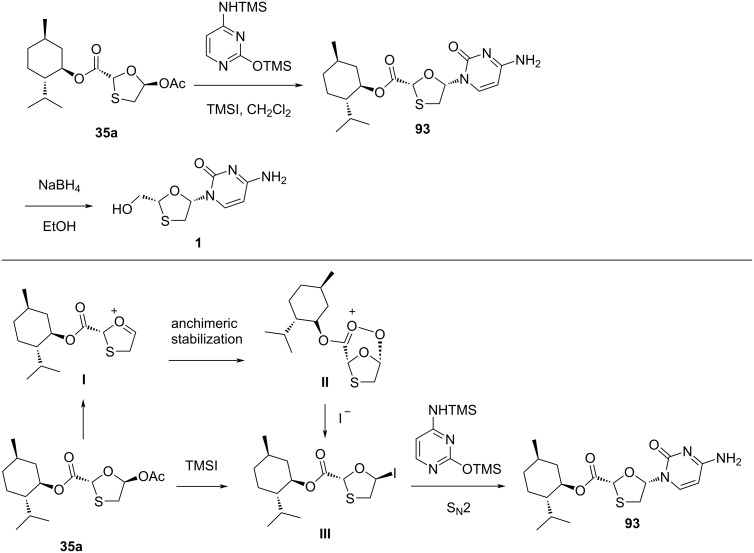
Stereoselective N-glycosylation for the synthesis of **1** by anchimeric assistance of a chiral auxiliary.

In 1995, a novel route was developed by Jin and co-workers [46], which utilized a Vorbrüggen N-glycosylation of the enantiomerically pure 5-acetoxyoxathiolane **35a** with presilylated cytosine as the key convergent step. This N-glycosylation reaction required the Lewis acid TMSI in a significant quantity to produce the desired cytidine **1** (Scheme 37).

As shown in a plausible mechanism in Scheme 37, it is assumed that 5-α-iodooxathiolane **III** is formed stereoselectively by reaction of the oxonium ion **I**, generated in situ by reaction of 5-acetoxyoxathiolane **35a** with TMSI, which stabilizes a C-2 ester substituent via anchimeric assistance (see **II**). The postulated mechanism shows that the iodide ion attacks on the stable oxonium ion to provide an anomer, which further reacts with the presilylated nucleobase in an S_N_2 manner and predominantly affords the β-cytidine adduct.

**Scheme 38:**
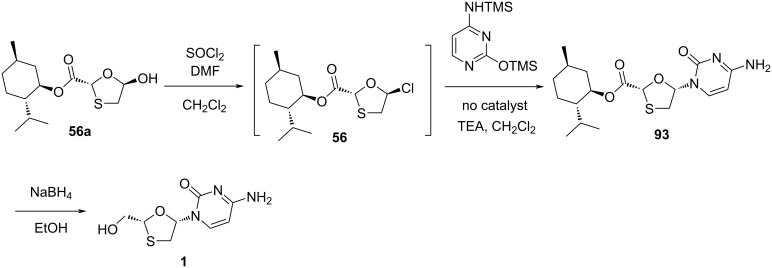
Whitehead and co-workers’ approach for the synthesis of **1** via direct N-glycosylation without an activator.

DKR overcomes the drawback of classical resolution since it is theoretically possible to obtain 100% yield of the desired isomer [74]. 5-Hydroxyoxathiolane intermediate **56a** was isolated in a DKR procedure by Whitehead and co-workers (Scheme 38) [55]. Further, 5-chlorooxathiolane **56** was isolated from chlorination reaction of 5-hydroxyoxathiolane **56a** using thionyl chloride in presence of catalytic DMF and dichloromethane solvent. This further reacted directly with the presilylated cytosine without any promoter or additive and gave nucleoside **93** in a selective manner (β/α 10:1). The ester group of nucleoside derivative **93** was further reduced with sodium borohydride in ethanol, which gave lamivudine (**1**). An efficient and enantioselective synthesis of lamivudine (**1**) was developed, which utilizes a highly effective DKR as the key step for obtaining pure substrate. The synthesis of **56a** via DKR was discussed earlier in this review (Scheme 17).

**Scheme 39:**
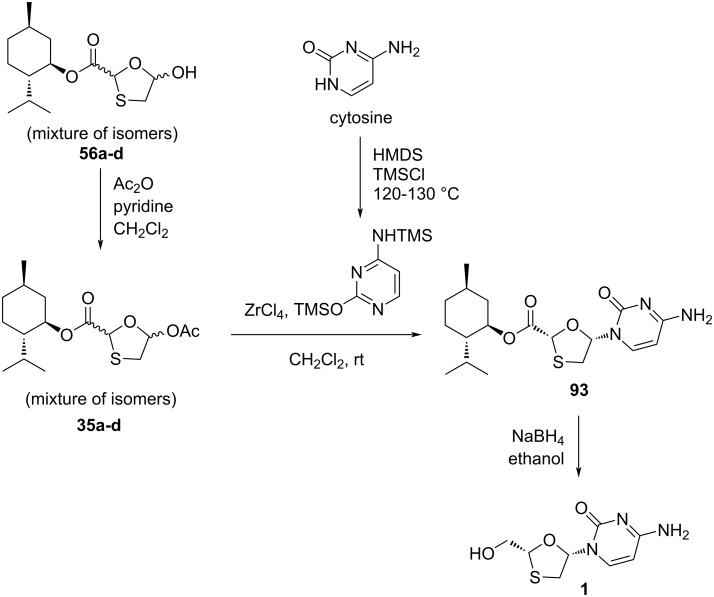
ZrCl_4_-mediated stereoselective N-glycosylation.

Recently, we have developed [75] an effective method for selective glycosylation using 0.5 equiv of ZrCl_4_ via the activation of oxathiolane acetates **35a**–**d**. The reaction was complete after a reduced reaction time and suitable for large-scale production with good yield at ambient temperature (Scheme 39). The usefulness of this method was that even without isolation of enantiomerically pure oxathiolane substrate, the facile stereoselective glycosylation took place and was improved compared to previously reported methods. The oxathiolane acetates **35a**–**d** were used in situ and stereoselectively led to a single nucleoside isomer **93**. After preparation of nucleoside ester intermediate **93**, lamivudine (**1**) was obtained with reducing agent sodium borohydride.

**Scheme 40:**
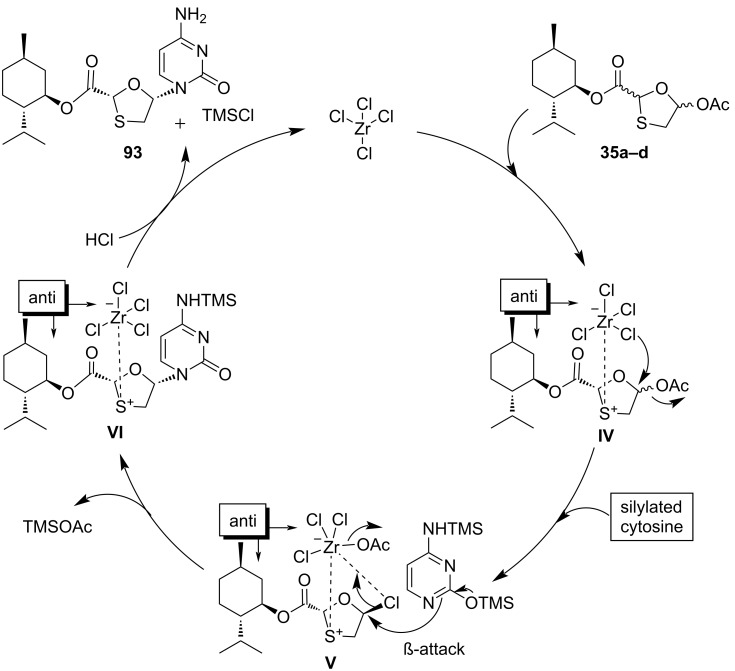
Plausible reaction mechanism for stereoselective N-glycosylation using ZrCl_4_.

The plausible reaction mechanism was also described for this selective N-glycosylation methodology (Scheme 40). A previously reported [72] plausible mechanism involving the use of SnCl_4_ was considered while proposing the mechanism when using ZrCl_4_ catalyst for the stereoselective N-glycosylation. We hypothesize that because of the Lewis acid character, ZrCl_4_ could most likely form a precomplex with the sulfur atom of the oxathiolane ring, as in **IV**. The presence of the chiral ʟ-menthyl ester auxiliary function assists the complexation with ZrCl_4_ in a specific orientation and could minimize the destabilization through 1,2-steric interactions. Therefore, the selectivity could herein be accomplished by means of anchimeric assistance by the ʟ-menthyl ester. Additionally, formation of intermediate **V** probably occurred due to the attack of one chloride ion on the anomeric carbon atom while maintaining α-configuration and simultaneous elimination of an acyl group as illustrated in intermediate **IV**. Further, attack of silylated cytosine on α-chloro-substituted derivative **V** in an S_N_2 reaction results in the formation of a glycosidic C–N bond in the β-configured nucleoside intermediate **VI**. In the last step, addition of HCl easily deprotects the TMS group of intermediate **VI** and affords compound **93** through simultaneous removal of ZrCl_4_. This approach of in situ precomplexing disallows the α-face attack of silylated cytosine.

**Scheme 41:**
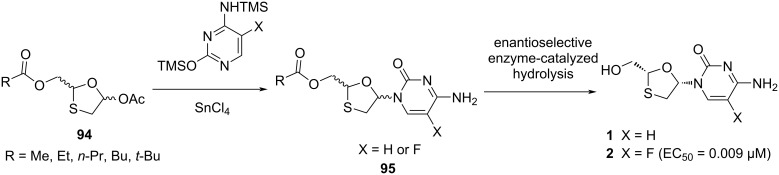
Synthesis of enantiomerically pure oxathiolane nucleosides **1** and **2**.

Liotta and co-workers [76] established an enzyme-catalyzed hydrolysis of protected racemic nucleosides to synthesize the enantiomerically pure oxathiolane nucleoside analogues **1** and **2** (Scheme 41). The protected racemic nucleoside derivatives **95** were synthesized by tin-mediated N-glycosylation of the corresponding acetate precursor **94** with silylated cytosine or 5-fluorocytosine. Further, hydrolysis of the 5'-*O*-acetyl group was evaluated with respect to reactivity and enantioselectivity utilizing several enzymes. They found that the butyrate ester derivative was hydrolyzed with a higher rate than the 5'-*O*-acetate derivative during the synthesis of ʟ-(−)-2',3'-dideoxy-5-fluoro-3'-thiacytidine (**2**). However, hydrolysis was observed to occur with a comparable rate to that of the 5'-*O*-valerate and 5'-*O*-propionate esters. Additionally, the rate of hydrolysis for the ester derivatives of FTC (**2**) was significantly higher than for the corresponding 3'-thiacytidine derivatives.

**Scheme 42:**
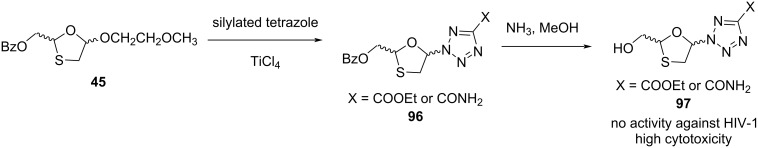
Synthesis of tetrazole analogues of 1,3-oxathiolane nucleosides **97**.

The tetrazole analogues of 1,3-oxathiolane nucleosides were synthesized by Faury et al. [50]. N-Glycosylation of silylated tetrazole with 1,3-oxathiolane precursor **45** in the presence of titanium tetrachloride or TMSOTf, followed by deprotection in methanolic ammonia gave the final nucleoside **97** (Scheme 42). Unfortunately, the introduction of a tetrazole ring to the oxathiolane moiety did not result in any anti-HIV activity and higher cytotoxicity.

**Scheme 43:**
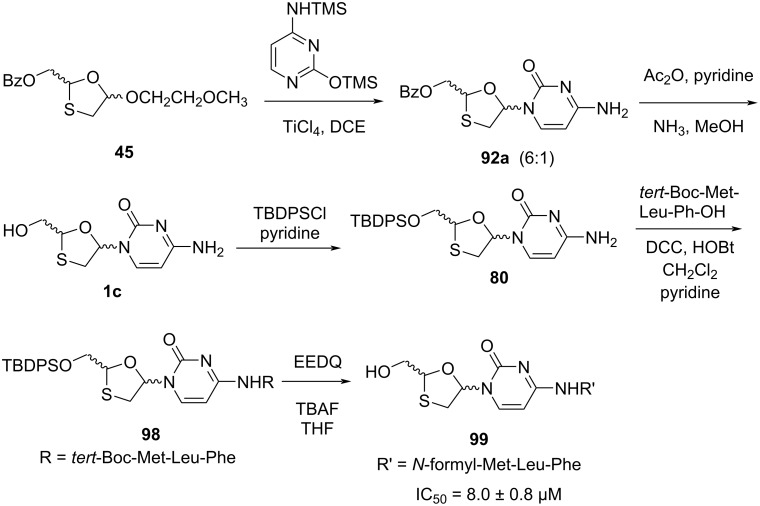
Synthetic approach toward **99** from 1,3-oxathiolane **45** by Camplo et al*.*

The synthesis of N^4^-substituted analogue **99** of 2',3'-dideoxy-3'-thiacytosine was discovered by Camplo et al*.* (Scheme 43) [77]. The prodrug was devised for targeting specific receptors on the leukocytes membrane. The crucial N-glycosylation reaction between 1,3-oxathiolane precursor **45** and silylated cytosine was carried out using TiCl_4_ as a catalyst. The N-acylation of compound **92a** was performed for flash chromatography, and further ammonolysis in methanol affords compound **1c**. The silylation of **1c** with TBDPSCl was carried out, and then coupling reaction with *tert*-Boc-Met-Leu-Phe-OH in the presence of DCC and HOBt provided compound **98**. The *tert*-Boc protecting group was further removed in formic acid, and the resulting nucleoside peptide was formylated using 2-ethoxy-1-ethoxycarbonyl-1,2-dihydroquinoline (EEDQ) as formylating reagent. Finally, the *tert*-butyldiphenylsilyl (TBDPS) group was desilylated using TBAF in THF solvent, which gave compound **99**. For compound **99**, the IC_50_ value of HIV-I cytopathogenicity in MT-4 cells was 8.0 µM at a concentration nontoxic to the host cells.

**Scheme 44:**
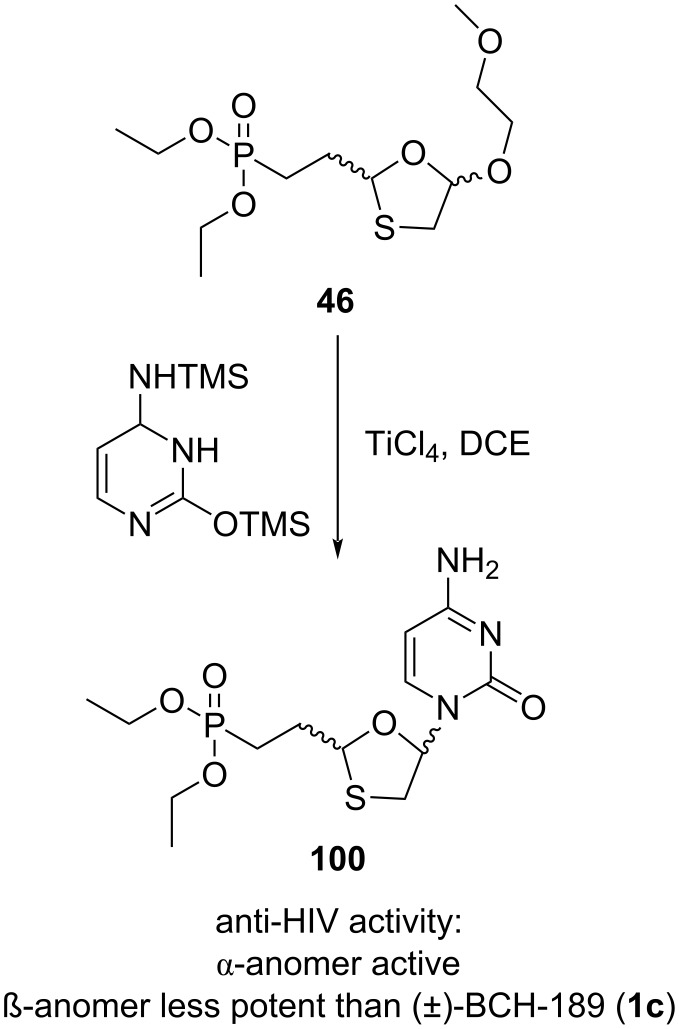
Synthesis of **100** from oxathiolane phosphonate analogue **46**.

In 1993, Kraus [51] developed the phosphonate analogue **100** of 3'-thia-2',3’-dideoxycytidine. The Lewis acid-mediated N-glycosylation reaction of the phosphonate analogue **46** of an oxathiolane precursor with an appropriate nucleobase afforded the phosphonate analogue **100** (Scheme 44). To obtain both the α- and β-anomers for biological assessment, TiCl_4_ was used as a Lewis acid in the glycosylation procedure in place of SnCl_4_. Separation of the α- and β-anomers was carried out after N^4^-acetylation by using acetic anhydride in DMF. The pure isomers were isolated in 80% yield in a 1:1 ratio. The phosphonate nucleosides were isolated by hydrolysis of the phosphonic acid ethyl ester, followed by treatment with methanolic ammonia. Anti-HIV assessment of these analogues demonstrated that the α-anomer was not active, while the β-anomer was less potent than the parent compound (±)-BCH-189 (**1c**). This could be because the phosphorylated modified analogue **100** was not a proper substrate for nucleotide kinases.

**Scheme 45:**
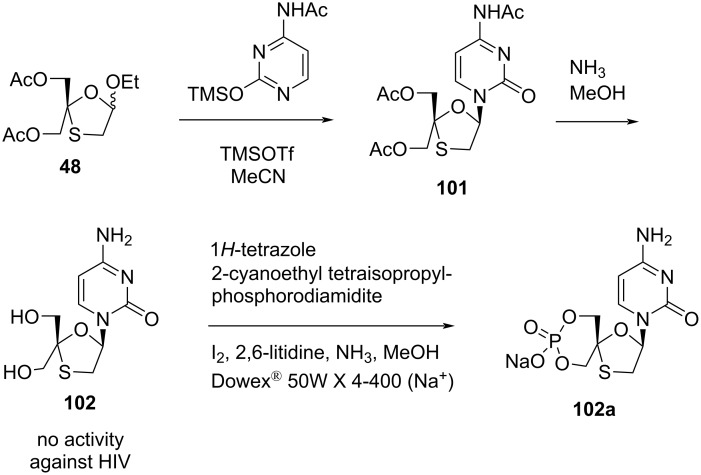
Synthetic approach toward **102** and the corresponding cyclic thianucleoside monophosphate **102a** by Chao and Nair.

1,3-Oxathiolane derivative **48** was glycosylated directly with persilylated *N*-acetylcytosine to provide nucleoside **101**, which gave nucleoside **102** after deprotection (Scheme 45). Thymine was also used instead of *N*-acetylcytosine, which gave the corresponding thymine-based nucleoside derivative. These were also converted to the spirocyclic monophosphate nucleosides **102a**, but none of the synthesized compounds showed anti-HIV activity. This study was performed by Chao and Nair in 1997 [52]. The procedure synthesized a racemic 4'-hydroxymethylated 2',3'-dideoxy-3'-thianucleoside analogue starting from compound **48** via N-glycosylation with silylated nucleobase in the presence of Lewis acid in acetonitrile solvent. Further deacetylation was carried out in methanolic ammonia to afford nucleoside **102**. Cyclic thianucleoside monophosphate **102a** was synthesized when nucleoside **102** was treated with 2-cyanoethyl tetraisopropylphosphorodiamidite in the presence of 1*H*-tetrazole in DMF, followed by oxidation with iodine and deprotection with methanolic ammonia.

**Scheme 46:**
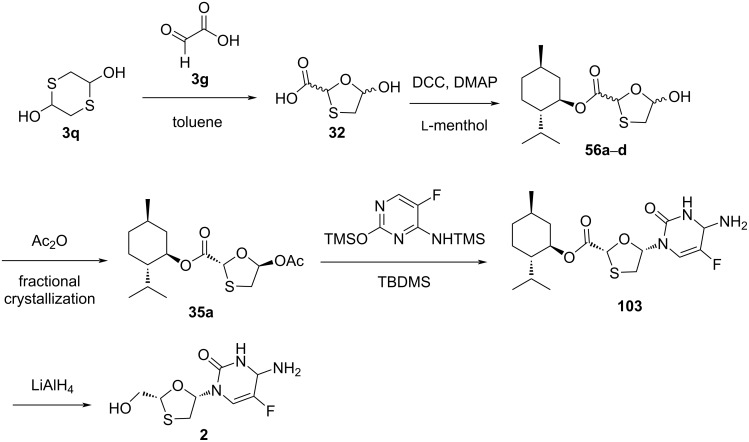
Synthesis of emtricitabine (**2**) from 1,4-dithiane-2,5-diol (**3q**) and glyoxylic acid (**3g**).

Mansour et al*.* [78] described a highly diastereoselective processes for producing *cis*-nucleoside analogues and derivatives in high optical purity. The oxathiolane derivative **32** was synthesized by the reaction of 1,4-dithiane-2,5-diol (**3q**) with glyoxylic acid (**3g**). Further, ʟ-menthol as a chiral auxiliary was introduced using DCC and DMAP, which gave *cis*- and *trans*-esters **56a**–**d** as a diastereomeric mixture (Scheme 46). The glycosylation reaction of **35a** with presilylated 5-fluorocytosine, followed by ester group reduction of **103** using LiAlH_4_, provided emtricitabine (**2**). The procedure illustrates the advantages of generating nucleosides of which the configuration can easily be controlled by the selection of the appropriate starting material.

**Scheme 47:**
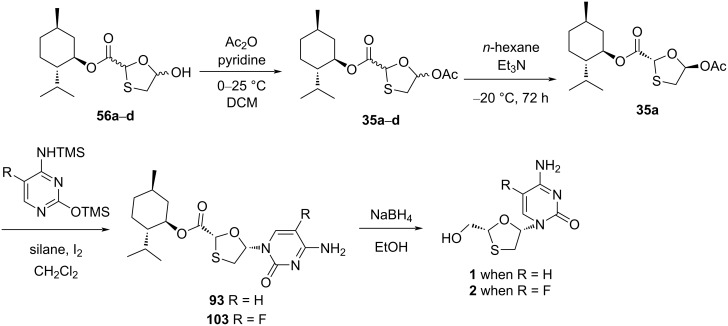
Synthesis of **1** and **2**, respectively, from **56a**–**d** using iodine-mediated N-glycosylation.

**Scheme 48:**
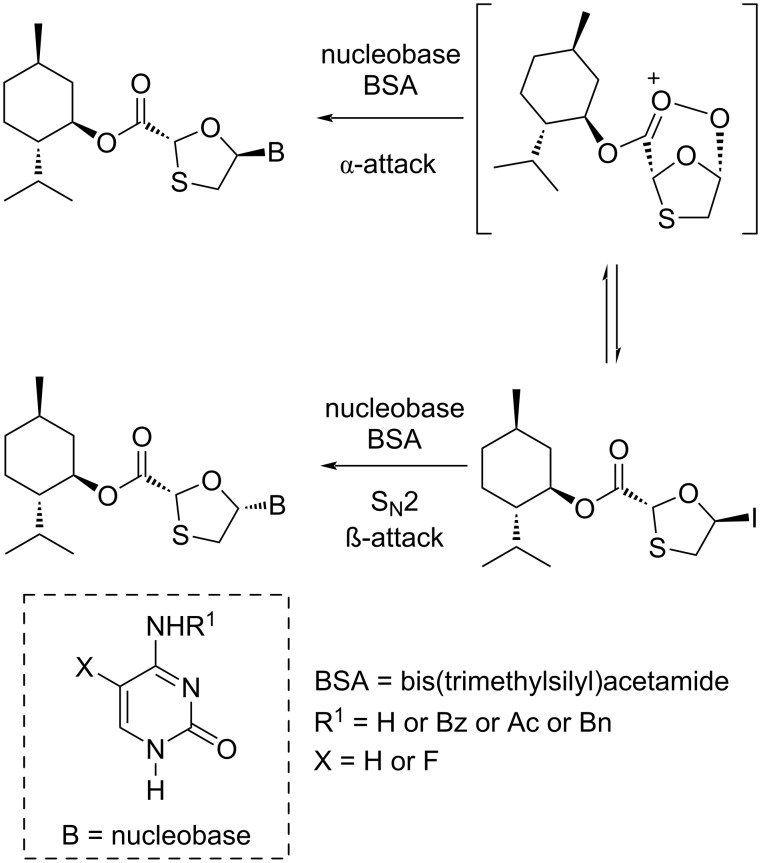
Plausible mechanism for silane- and I_2_-mediated N-glycosylation.

The silanes Et_3_SiH and PMHS, respectively, were used along with I_2_ as novel N-glycosylation reagents for the synthesis of 3TC (**1**) and FTC (**2**), as reported by Caso et al. [79]. These systems were developed to promote the substrate N-glycosylation. The enantiopure 1,3-oxathiolane acetate **35a** was isolated from *n*-hexane and TEA at −20 °C after stirring for about 72 h. This intermediate was further reacted with a silylated cytosine derivative via N-glycosylation, which afforded the nucleoside analogues **93** and **103**, respectively. Stereoselectivity was achieved in the reactions, and the stereochemical outcome of the reaction was influenced by the nature of the protecting group at position N^4^ of 5-fluorocytosine (Scheme 47). This method was reasonably considered as an effective alternative to the available procedures because of the use of inexpensive and more stable reagents. An important role in determining the stereochemical outcome was played by the N^4^-protecting group of 5-fluorocytosine, presumably based on the capacity to increase the soft character of the nucleobase. A possible mechanism was also provided (Scheme 48), in which the chiral auxiliary ʟ-menthol assists the selective β-nucleoside formation. The ester functionality of the nucleoside derivatives **93** and **103** was easily converted to a primary hydroxy group upon reduction with sodium borohydride in ethanol, which gave **1** and **2**, respectively.

**Scheme 49:**
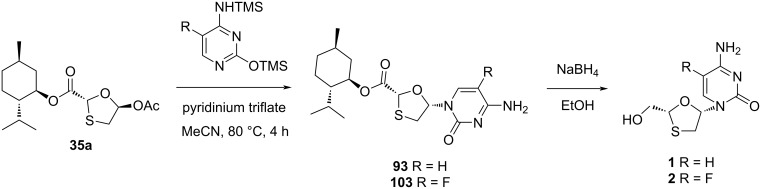
Pyridinium triflate-mediated N-glycosylation of **35a**.

Mandala and Watts [80] reported the first use of pyridinium triflate as a novel N-glycosylation reagent for the synthesis of the antiviral drugs lamivudine (**1**) and emtricitabine (**2**, Scheme 49). The key 5-acetoxyoxathiolane intermediate **35a** was prepared in high yield by a catalyst- and solvent-free method within a minimum reaction time. Further, a greener procedure by using sodium bicarbonate as the base for the acetylation reaction instead of pyridine was implemented to prepare **35a**.

**Scheme 50:**
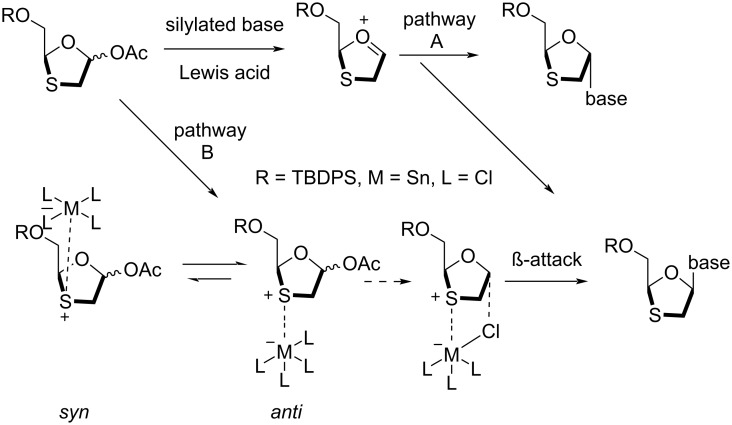
Possible pathway for stereoselective N-glycosylation via in situ chelation with a metal ligand.

In the 1990s, Liotta, Choi, and co-authors [18,72] reported a highly stereoselective N-glycosylation reaction that was controlled via in situ chelation of the oxathiolane moiety and an appropriate Lewis acid (Scheme 50). The exclusive formation of the β-anomer of a precursor of lamivudine (**1**) was achieved by the use of stannic(IV) chloride in dichloromethane solvent at ambient temperature. This way, the stereochemistry in the N-glycosylation reaction is predictable. The stereoselectivity in the N-glycosylation reaction could be organized based on a preferential interaction between the sulfur heteroatom and an appropriate Lewis acid. The use of the Lewis acids TMSOTf and TMSI generates an oxonium ion, which reacts further following pathway A in Scheme 50, and hence no stereocontrol was found in the resultant product. But when the Lewis acid precomplexed the sulfur heteroatom of the ring, selectivity in a diastereofacial manner could be achieved (i.e., pathway B) by complexation *anti* to the protected hydroxymethyl substituent. This complexation may restrict the orientation of the attack of the nucleobase moiety to the α-face. The metal that provides a chloride ligand to the α-face of the oxonium ion could possibly undergo S_N_2 attack, and hence the formation of the β-N-nucleoside resulted.

**Scheme 51:**
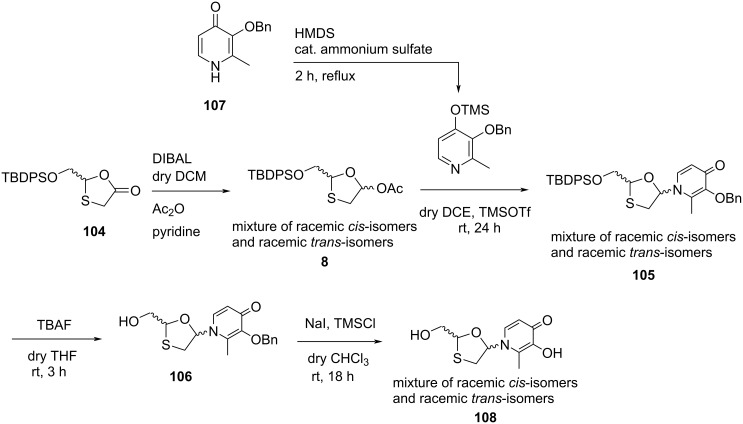
Synthesis of novel 1,3-oxathiolane nucleoside **108** from oxathiolane precursor **8** and 3-benzyloxy-2-methylpyridin-4-one (**107**).

Barral and co-workers [81] synthesized cyclic 2′,3′-dideoxynucleoside compounds in which a 3-hydroxy-2-methylpyridin-4-one species was used as the nucleobase. The synthesized nucleosides **108** contain sugar moieties similar to the oxathiolane nucleosides, namely 3TC (**1**). The heterocyclic base 3-benzyloxy-2-methylpyridin-4-one (**107**) was silylated using HMDS in the presence of catalytic ammonium sulfate. The reaction further involved conventional N-glycosylation with the oxathiolane precursor **8** in 1,2-dichloroethane using TMSOTf as a catalyst. As oxathiolane precursor **8** was sterically impurely obtained from racemic thialactone **104** after reduction with DIBAL and subsequent acetylation, **105** was formed as a mixture of racemic *cis*-nucleosides and racemic *trans*-nucleosides after N-glycosylation. The TBDPS group of these nucleosides was further deprotected using TBAF in THF. Since the Pd catalyst is poisoned due to the sulfur present in the oxathiolane ring, further debenzylation of the nucleobase was achieved by using in situ-generated trimethylsilyl iodide, which gave final **108** as a mixture of racemic *cis*-nucleoside and racemic *trans*-nucleoside (Scheme 51).

**Scheme 52:**
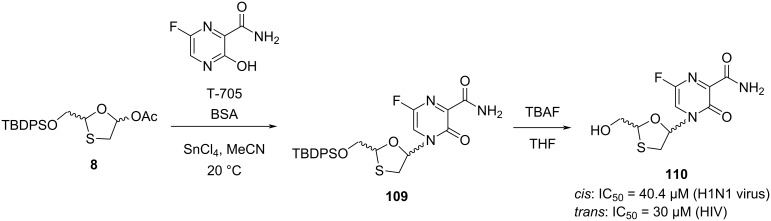
Synthesis of **110** using T-705 as a nucleobase and 1,3-oxathiolane derivative **8** via N-glycosylation.

A novel class of 1,3-oxathiolane nucleoside derivatives of favipiravir (T-705) was synthesized and investigated recently by Han et al. Some of the analogues were found to have good anti-HIV and anti-H1N1 activity [43]. The N-glycosylation reaction of 1,3-oxathiolane derivative **8** with a novel nucleobase, which is known as T-705, was carried out. Firstly, silylation of nucleobase T-705 was performed in a BSA and acetonitrile mixture. The N-glycosylation was accomplished using SnCl_4_ catalyst (Scheme 52), providing nucleoside **109**, which was further converted to the nucleoside **110** using TBAF in THF. The nucleosides analogue **110** formed as a mixture of *cis*- (45%) and *trans*-isomers (50%). Interestingly, the *cis*-isomer showed activity against the H1N1 influenza virus (IC_50_ = 40.4 µM), while the *trans*-isomer showed weak activity against HIV (IC_50_ = 30 µM).

**Scheme 53:**
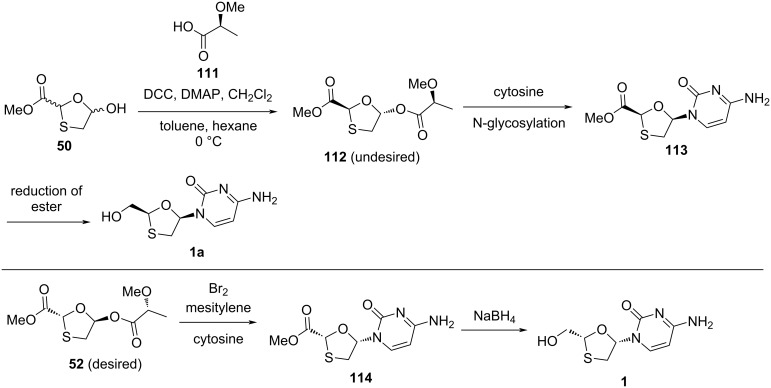
Synthesis of **1** using an asymmetric leaving group and N-glycosylation with bromine and mesitylene.

Snead and co-workers [53] recently developed a new approach for stereoselective nucleoside synthesis that enables a cost-effective approach to lamivudine (**1**, Scheme 53). The synthesis of lamivudine (**1**) was established by employing a method that defines the stereochemistry at the oxathiolane moiety. For this, a commercially available lactic acid derivate **111** served a dual purpose, namely the activation of the anomeric center for N-glycosylation and the transfer of the stereochemical information to the substrate. The enantiomers of the lactic acid derivative **111** are available and used to access the β-enantiomer. The research group also discovered that an asymmetric leaving group was useful for acylation in a selective manner by directing the absolute stereochemistry of the resultant nucleoside, and it provides reliable access to either enantiomer. The acylation of 1,3-oxathiolane **50** with (*S*)-lactic acid derivative **111** and further crystallization in toluene/hexane at 0 °C provided a single isomer **112**. However, compound **112** did not have the desired configuration. Therefore, while using the compound **112**, the undesired 3TC-derived enantiomer **1a** may end up as a final product via N-glycosylation, followed by reduction of the ester group to the primary hydroxy group. Considering this proof of concept, the research group used the other isomer **52** to access the desired configuration of 3TC (**1**). The synthesis was a high-yielding linear four-step sequence that made use of inexpensive raw materials. Also, the use of low-molecular-weight intermediates efficiently increased the material throughput, setting the stage for reduced costs of goods derived from 3TC (**1**). For the N-glycosylation reaction, bromine and mesitylene reagents were used, which generated HBr, and hence acylated oxathiolane **52** was quantitatively transformed in situ to the brominated analogue, which acted as an active precursor to the nucleoside. Then, coupling with the nucleobase cytosine resulted in the formation of nucleoside **114** in good yield. Further, removal of the ester group of **114** using sodium borohydride afforded 3TC (**1**).

### Efforts for the separation of racemic mixtures of 1,3-oxathiolane nucleosides

Biological activities of nucleosides generally reside in a single enantiomer, and enzymes are often used for the resolution of racemic nucleosides [82]. To understand which of the enantiomers of a nucleoside has potential antiviral activity, scientist have separated the enantiomers with a variety of methods, such as chiral HPLC as well as enzymatic and chemical methods for the determination of the anti-HIV activity and cytotoxicity in vitro. Coates et al*.* [83] made efforts for the separation of enantiomers of racemic (±)-BCH-189 (**1c**) with a chiral HPLC method, and it was accomplished using a column known as Cyclobond I acetyl (acetylated β-cyclodextrin). This section reviews the enzymatic as well as the chemical methods used to separate a racemic mixture of 1,3-oxathiolane nucleosides.

#### Enzymatic methods

**Scheme 54:**
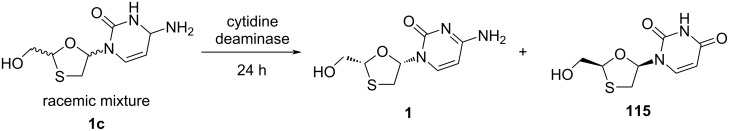
Cytidine deaminase for enzymatic separation of **1c**.

The use of enzymes for the resolution of racemic compounds is widespread, and enzymes have been used frequently in the synthesis of nucleosides. The synthesis of optically pure 3TC (**1**) by utilizing enzymatic resolution was also established by Mahmoudian et al. [84]. Cytidine deaminase from *Escherichia coli* deaminated only the ᴅ-form of 2'-deoxy-3'-thiacytidine, which converted **1c** to compound **115**, leaving the optically pure ʟ-form 3TC (**1**) unreacted (Scheme 54). The cytidine deaminase EC 3.5.4.5 from *Escherichia coli* easily deaminated 2’-deoxy-3’-thiacytidine in an enantioselective manner and produced optically pure 3TC (**1**).

**Scheme 55:**
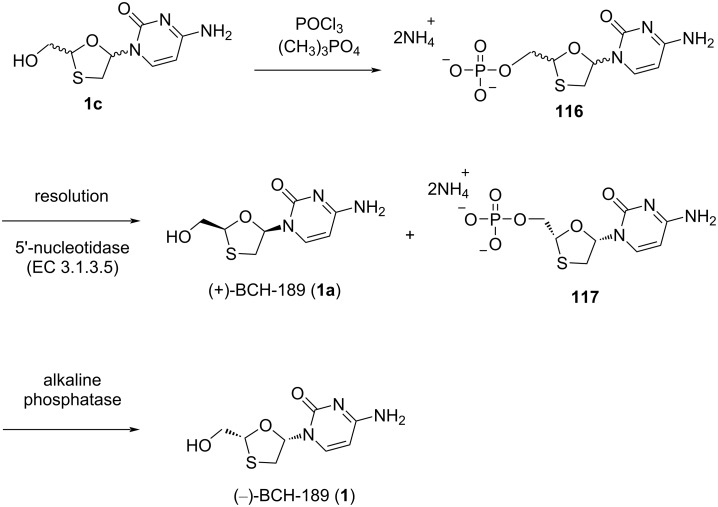
Enzymatic resolution of the monophosphate derivative **116** for the synthesis of (−)-BCH-189 (**1**) and (+)-BCH-189 (**1a**).

The enzymatic resolution of the monophosphate derivative **116** of (±)-*cis*-[2-(hydroxymethyl)-1,3-oxathiolan-5-yl]cytosine using the 5'-nucleotidase from *Crotalus atrox* venom allowed facile access to the individual enantiomers, which was reported by Storer et al. [24]_._ The racemic mixture **1c**, upon treatment with phosphorous oxychloride in the presence of trimethyl phosphate at a temperature 0 °C and further appropriate work-up, produced a racemic monophosphate as the ammonium salt **116**. Later, a solution of the racemic monophosphate **116** in an aqueous buffer at 37 °C was prepared from glycine and magnesium chloride upon treatment with 5'-nucleotidase (EC 3.1.3.5), which resulted in a two-component mixture. This was further separated by chromatography, which gave enantiomerically pure (+)-BCH-189 (**1a**) and the monophosphate **117** of (−)-BCH-189 (**1**). The product was then dephosphorylated by an alkaline phosphatase to afford (−)-BCH-189 (**1**, Scheme 55).

**Scheme 56:**
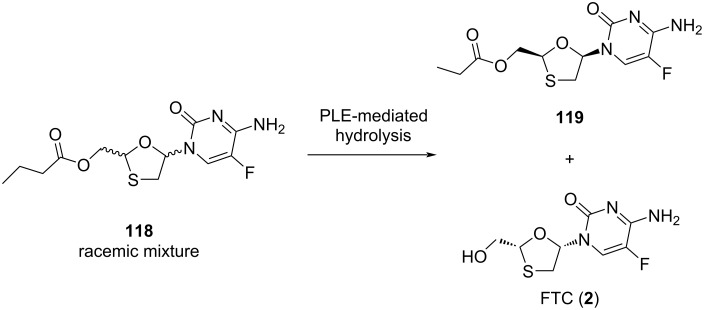
Enantioselective resolution by PLE-mediated hydrolysis to obtain FTC (**2**).

Liotta et al. [76] reported an approach for the highly enantioselective resolution to obtain emtricitabine (**2**) as well as related sulfur-containing nucleosides with enzyme catalysis, which uses a PLE-mediated hydrolysis procedure of butyrate ester derivative **118**. The use of the butyrate ester selectively separated the unreacted substrate **119** from the medium by an extraction procedure with chloroform. This process was developed to the synthesis of enantiomerically pure **2** in a gram quantity (Scheme 56).

#### Chemical methods

The choice of a proper resolving agent and an appropriate crystallization solvent are the two determining factors for the successful resolution of enantiomers.

**Scheme 57:**
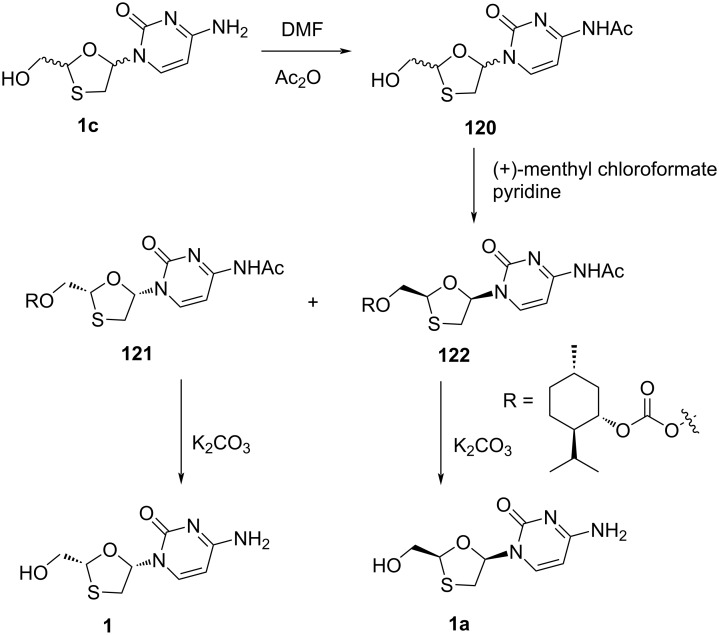
(+)-Menthyl chloroformate as a resolving agent to separate a racemic mixture **120**.

In 2002, Li et al. [85] described the chemical resolution of a racemic mixture of lamivudine (**1**) and **1a** using chiral resolving agents, such as (−)-camphanic acid chloride and (+)-menthyl chloroformate. Out of these two, (+)-menthyl chloroformate was used as a promising resolving agent to separate racemic (±)-BCH-189 (**1c**). The primary amine group was initially protected by acetylation using acetic anhydride in DMF. Further, the corresponding acetyl derivative **120** was reacted with (+)-menthyl chloroformate in the presence of pyridine, providing a mixture of the diastereomers **121** and **122**. The crystallization of these diastereomers in methanol at 0 °C afforded the (−)-diastereomer, while the (+)-diastereomer was isolated by concentration, followed by recrystallization from mother liquor. Further, separate deprotection of the diastereomers with potassium carbonate gave the (−)-enantiomer lamivudine (**1**) and the opposite (+)-enantiomer **1a** (Scheme 57).

Through using chiral host compounds, such as dinaphthalenephenols (e.g., BINOL), diphenanthrenols, or tartaric acid derivatives, Deng and co-workers [86] reported the resolution of prazoles. The resolution approach resulted in the formation of a 1:1 of the complex, involving the chiral host and the desired enantiomer as a guest molecule, while the undesired enantiomer remained in solution. (*S*)-Omeprazole, a potent inhibitor of gastric acid secretion, has been isolated in pure form from a racemic mixture by using this chiral host–guest method involving (*S*)-(–)-BINOL.

**Scheme 58:**
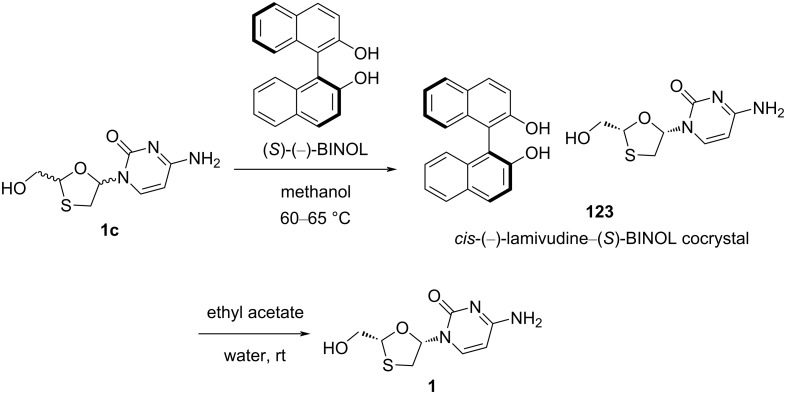
Separation of racemic mixture **1c** by cocrystal **123** formation with (*S*)-(−)-BINOL.

In 2009, we demonstrated a chemical resolution process for racemic mixture **1c** consisting of lamivudine (**1**) and **1a** by forming cocrystal with (*S*)-(−)-BINOL (Scheme 58) [87]. Lamivudine (**1**) was obtained in high purity and more than 99.9% ee. Interestingly, it was found that the *cis*-(−)- and *trans*-(−)-enantiomers also formed cocrystals with (*S*)-(−)-BINOL, leaving behind the *cis*-(+)- and *trans*-(+)-isomers in the solution. It is worth to mention that the four stereoisomers based on the lamivudine core structure were also separated using this strategy. These isomers were further isolated and characterized. The racemic mixture **1c**, consisting of lamivudine (**1**) and **1a**, was mixed with (*S*)-(−)-BINOL in methanol at reflux temperature. The *cis*-(−)-lamivudine–(*S*)-BINOL complex **123** was isolated at room temperature by filtration. The *cis*-(−)-lamivudine–(*S*)-(−)-BINOL complex **123** was further dissolved in a water/ethyl acetate system where (*S*)-(−)-BINOL was extracted by ethyl acetate and lamivudine (**1**) remained in water.

Overall, over decades, numerous procedures have been utilized to synthesize 1,3-oxathiolane nucleosides. However, from an industrial perspective, asymmetric procedures are more viable because they are more efficient with respect to the cost and atom economy. Attempts are currently being made to develop cost-effective, simpler, and atom-economic processes for these nucleoside analogues. Glycosylation reactions where the formation of the C–N bond to the anomeric center determines the stereochemistry of the resultant product are crucial. The use of enzymes for these syntheses have also been shown to be an alternative to existing chemical methods. However, the use of enzymes in industry is somewhat difficult to implement, but it is being developed nonetheless because of the current interest in sustainable chemistry. As a result, combined chemoenzymatic procedures can be recognized as a viable alternative to the conventional synthesis for such a type of modified nucleosides.

## Conclusion

Invention and improvement of 1,3-oxathiolane nucleosides is indisputably one of the important success stories of recent research in nucleoside chemistry. In the last three decades since Belleau et al. [38] produced the first oxathiolane nucleoside as racemic (±)-BCH-189 (**1c**), significant contributions from different research groups to the stereoselectivity, structural modification, and reactivity of these analogues have led to applications as a variety of therapeutic agents. Due to the biological significance of these nucleosides, efficient synthetic routes with stereocontrol and high yield are in great demand. The heterogeneity of oxathiolane nucleoside structures, along with the unpredictable outcome of the glycosylation process, which depends on the reaction conditions, is influenced by the nature of the glycosyl accepter substrate, catalyst, type of leaving group, and protecting groups, makes it a more daunting task.

However, as we anticipate from the recent research discussed in this review, the efforts made to access such nucleoside analogues are inadequate. One promising area is the field of chiral auxiliaries or Lewis acid catalysis, where the strength of the coupling of the nucleobase with the sugar can allow for enhanced stabilization of oxonium ions, with the potential for stereoselectivity in N-glycosylation via in situ complexation or anchimeric assistance, a key step for nucleoside formation. Significant advances are also being made in fields where the use of enantiomerically pure oxathiolane precursors is more established. In this review, various methodologies to access 1,3-oxathiolane nucleoside analogues that are used to treat AIDS are reviewed. The efforts for the construction of many such nucleoside analogues require a selective glycosylation reaction, which is important to unite appropriate furanose sugar derivatives and nucleobases by forming C–N glycosidic bonds while maintaining selectivity.

In conclusion, we summarize that the desired stereoselectivity in 1,3-oxathiolane nucleoside synthesis was achieved by using i) asymmetric starting materials, ii) asymmetric leaving groups, iii) in situ chelation with appropriate activators or Lewis acids, iv) chiral-auxiliary-induced methods via anchimeric assistance, v) DKR methods, and vi) enzymatic or chemical resolution methods. This classification could be convenient for organic chemists and researchers working in the area of nucleoside research.

Regardless of all the global efforts, HIV/AIDS is still a chronic disease due to rapid mutations of the virus and more prevalent drug resistance. However, many antiviral drugs have been approved, particularly NRTIs, which in most cases can transform this disease from fatal to chronic. ʟ-Nucleosides represent a specific class of drugs with a better activity and low toxicity due to their specific interaction with reverse transcriptase rather than human DNA polymerase. Within this class of drugs, 1,3-oxathiolane nucleosides, such as 3TC (**1**) and FTC (**2**), are still the most representative FDA-approved medicines, indicating a low cytotoxicity and a potent activity both in vitro and in vivo conditions. Their synthesis is still a major concern for researchers, who aim for high yield and good stereoselectivity as well as an inexpensive and safe production. Indeed, a modern synthetic approach is being sought that can avoid the utilization of unstable promoters in N-glycosylation protocols and attain a superior selectivity [88]. More specifically, there is still a need to accelerate new research for discovering anti-HIV agents that feature novel mechanisms of action and can work against drug resistance phenomena. The significance of more creative and easy procedures to access not only 1,3-oxathiolane nucleosides but various nucleoside molecules with desired structural modifications is still a major challenge for synthetic chemists [89–90]. On the grounds of this, synthetic nucleoside analogues have found an application in rational biomolecular designing as well as in medicinal chemistry [90]. These modified 1,3-oxathiolane nucleosides could be transformed into oligonucleotides to investigate the potential to act as antisense nucleosides. Accordingly, because some of these nucleosides have demonstrated anti-HIV, anti-HBV, and anti-H1V1 activity, they could also be thoroughly investigated for further biological activity. This review will benefit researchers in understanding the various processes for synthesizing 1,3-oxathiolane nucleosides as well as their involvement in the chain termination process in medicinal chemistry. The chemists researching modified nucleosides will be encouraged to advance the access to unexplored 1,3-oxathiolane nucleosides and the use of various nucleobases, such as purines, pyrimidines, or their derivatives. Further, it appears promising to develop the stereoselective chemistry of nucleoside analogues to evaluate the resulting products as potential anti-HIV and anticancer drugs.
